# High-Resolution, 3D Imaging of the Zebrafish Gill-Associated Lymphoid Tissue (GIALT) Reveals a Novel Lymphoid Structure, the Amphibranchial Lymphoid Tissue

**DOI:** 10.3389/fimmu.2021.769901

**Published:** 2021-11-16

**Authors:** Alf S. Dalum, Aurora Kraus, Shanawaz Khan, Erna Davydova, Dimitri Rigaudeau, Håvard Bjørgen, Adrián López-Porras, Gareth Griffiths, Geert F. Wiegertjes, Erling O. Koppang, Irene Salinas, Pierre Boudinot, Julien Rességuier

**Affiliations:** ^1^ Nofima, Norwegian Institute of Food, Fisheries and Aquaculture Research, Ås, Norway; ^2^ Center for Evolutionary and Theoretical Immunology (CETI), Department of Biology, University of New Mexico, Albuquerque, NM, United States; ^3^ Department of Biosciences, FYSCELL, University of Oslo, Oslo, Norway; ^4^ Department of Biosciences, BMB, University of Oslo, Oslo, Norway; ^5^ INRAE, IERP, Université Paris-Saclay, Jouy-en-Josas, France; ^6^ Section of Anatomy, Faculty of Veterinary Medicine, Norwegian University of Life Sciences, Ås, Norway; ^7^ Aquaculture and Fisheries Group, Department of Animal Sciences, Wageningen University & Research, Wageningen, Netherlands; ^8^ INRAE, UVSQ, VIM, Université Paris-Saclay, Jouy-en-Josas, France

**Keywords:** ALT, GIALT, gills, ILT, immunology, lymphoid tissue, mucosal immunity, zebrafish

## Abstract

The zebrafish is extensively used as an animal model for human and fish diseases. However, our understanding of the structural organization of its immune system remains incomplete, especially the mucosa-associated lymphoid tissues (MALTs). Teleost MALTs are commonly perceived as diffuse and scattered populations of immune cells throughout the mucosa. Yet, structured MALTs have been recently discovered in Atlantic salmon (*Salmo salar L.*), including the interbranchial lymphoid tissue (ILT) in the gills. The existence of the ILT was only recently identified in zebrafish and other fish species, highlighting the need for in-depth characterizations of the gill-associated lymphoid tissue (GIALT) in teleosts. Here, using 3-D high-resolution microscopy, we analyze the GIALT of adult zebrafish with an immuno-histology approach that reveals the organization of lymphoid tissues *via* the labeling of T/NK cells with an antibody directed to a highly conserved epitope on the kinase ZAP70. We show that the GIALT in zebrafish is distributed over at least five distinct sub-regions, an organization found in all pairs of gill arches. The GIALT is diffuse in the pharyngeal part of the gill arch, the interbranchial septum and the filaments/lamellae, and structured in two sub-regions: the ILT, and a newly discovered lymphoid structure located along each side of the gill arch, which we named the Amphibranchial Lymphoid Tissue (ALT). Based on RAG2 expression, neither the ILT nor the ALT constitute additional thymi. The ALT shares several features with the ILT such as presence of abundant lymphoid cells and myeloid cells embedded in a network of reticulated epithelial cells. Further, the ILT and the ALT are also a site for T/NK cell proliferation. Both ILT and ALT show structural changes after infection with Spring Viraemia of Carp Virus (SVCV). Together, these data suggest that ALT and ILT play an active role in immune responses. Comparative studies show that whereas the ILT seems absent in most neoteleosts (“Percomorphs”), the ALT is widely present in cyprinids, salmonids and neoteleosts, suggesting that it constitutes a conserved tissue involved in the protection of teleosts *via* the gills.

## Introduction

Over the years, the zebrafish (*Danio rerio*) has become a prominent animal model to study biological mechanisms related to immunity and pathology of humans and fish. Thus, zebrafish are widely used as a model for numerous fields of biomedical research, including cancer (1–3), immunity (4, 5), and disease modeling (6–8).

The interest in the zebrafish model for immunology-related studies is supported by the conservation of key features between the immune system of teleosts and mammals (9–11). The major immune cell types described in mammals have also been found in zebrafish, including: neutrophils (12), mast cells (13), macrophages (14), dendritic cells (15, 16), T lymphocytes (17), B lymphocytes (18, 19), and innate lymphoid cells (20). Indeed, the zebrafish immune system relies on both innate and adaptive mechanisms, and pathways common to all jawed vertebrates (21–24). However, there also are fundamental differences relevant to immunity between fish and mammals such as the lack of lymph nodes and tonsils in teleosts (25). The characterization of the zebrafish immune system has progressed rapidly during the last decade at both the cellular and molecular levels (26–28), however, the understanding of the structural organization of the zebrafish immune system, especially at the adult stage, remains incomplete.

The adult zebrafish has two primary known lymphoid organs: the kidney (mainly the pronephros or “head-kidney”), where hematopoietic stem cells and B cell precursors reside, and the thymus for T cell development. Although the kidney also act as a secondary lymphoid organ, the spleen is considered as the main true secondary organ (25). Lymph nodes, germinal centers and tonsils have not been identified, yet, inducible structures can form upon immune stimulation such as granulomas (29) and melano-macrophage centers (30–33). Zebrafish mucosal barriers are first lines of defense and the home to mucosa-associated lymphoid tissues (MALTs) (34, 35) among which are the gut-associated lymphoid tissue (GALT), the nasal-associated lymphoid tissue (NALT), the skin-associated lymphoid tissue (SALT) and the gill-associated lymphoid tissue (GIALT). In mammals, these tissues have a well-defined spatial organization that includes organized-MALT (O-MALT) structures. In teleost fish, MALTs are thought to consist of non-organized leukocytes scattered throughout the mucosal tissues, hence forming only a diffuse MALT (d-MALT) (23, 34, 36). D-MALT is thought to be present in all vertebrates, from agnathans to mammals, whereas bona fide O-MALT structures that support germinal center reactions are restricted to endotherms (birds and mammals) (37–39). An intermediate degree of lymphoid organization at mucosal barriers is found in Sarcopterygian fishes (lungfish) and amphibians, where lymphoid aggregates without distinct B and T cell zones are present at several mucosal tissues (40, 41). However, the absence of O-MALT structures in teleosts has been challenged by the identification of a lymphoid tissue structured by reticulated epithelial cells in the gills of Atlantic salmon (*Salmo salar L.*), called the “Interbranchial lymphoid tissue” (ILT) (42, 43). This structure proved also to be present in a number of other teleost species, including the zebrafish (44). This finding underlines the need to improve our knowledge of the lymphoid organization of zebrafish gills. The identification of the ILT, in addition to the discovery of the salmonid bursa (45), will possibly induce modifications of the commonly used terminology for mucosal immune tissues in fish, but in this paper, we will follow the conventional terminology.

The gills are the main respiratory organ in fish, providing a structure for gas exchange between the blood and the surrounding environment. Gills are also involved in maintaining osmolarity homeostasis, ion-regulation, acid-base regulation, excretion of nitrogenous waste, and the sense of taste *via* the presence of taste buds (46). Facilitating these functions, the gill mucosa forms a large surface area of interaction with the environment, but at the price of an increased exposure to foreign elements such as food particles, environmental debris, and pathogens. The classical overall anatomy of fish gills, its epithelium and its vasculature, has been extensively described by Wilson, Laurent and Olson (47–49) [see (50) for zebrafish]. In zebrafish, the gills are composed of 4 pairs of gill arches, located within cavities placed on each side of the head, called gill chambers. The gill arches bridge the sub-pharyngeal region and the roof of the gill chamber on a rostro-caudal axis. The gill arch represents a structural support for the gills, enclosing the high-pressure blood vessels (afferent arch artery and efferent arch artery) that originate from the ventral aorta.

**Figure 1:**
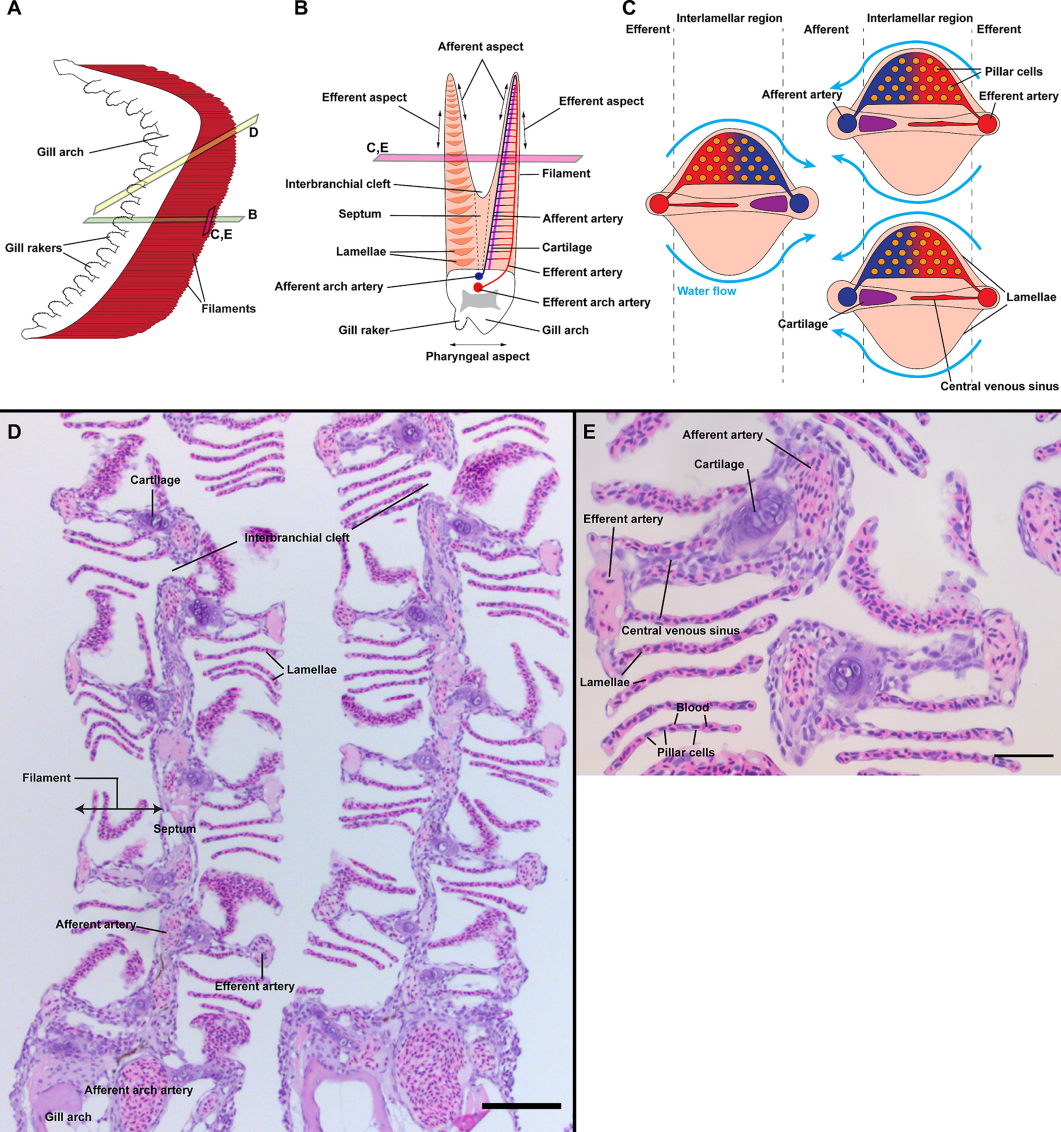
General organization of zebrafish gills. Schematic representations of the overall anatomy of adult zebrafish gills as observed from the side **(A)**, through a transversal plane across the gill arch **(B)** and through a coronal plane across gill filaments **(C)**. Section plane are illustrated in **(A, B)**. The blood flows from the afferent artery to the lamellae where it gets oxygenated and leave by the efferent artery. Counter-current to the blood, the water irrigates the lamellae from the efferent aspect to the afferent aspect of filaments. **(D)** Paraffin section (2 µm) displaying two gill arches through an oblique coronal plane, as illustrated in **(A)**. **(E)** Paraffin section (2 µm) displaying two filaments through a coronal plane, as illustrated in **(A, B)**. Scale bars: 50 µm **(D)** and 20 µm **(E)**.

From the pharyngeal side of the gill arch emanate cartilaginous structures, called gill rakers, which trap larger objects before they may harm the respiratory epithelium. This pharyngeal aspect, or medial side, of the gills is covered by a multilayered epithelium interspersed with occasional taste buds. From the lateral side of the gill arch, opposite to the pharyngeal side, two rows of arborescent structures called ‘filaments’ (or primary lamellae) emerge. In most “basal” teleost groups, such as salmonids and cyprinids, these two rows of filaments are separated by a supportive interbranchial septum that is mostly absent in neoteleosts (51). Throughout the length of the filaments arise a multitude of winged-shaped structures called lamellae (or secondary lamellae), representing the “branches” of the filaments. These lamellae are the sites where exchanges between blood and water occur, a process facilitated by the thin squamous epithelium that covers them. Filaments are subdivided into the afferent aspect for the inner side, the efferent aspect for the outer side, and the interlamellar region for the region in-between. Blood flows into the lamellae from the afferent aspect of the filament and exits through the efferent aspect; this runs counter-current to the flow of water circulating through the gill chamber during respiration. The overall anatomy of gills is illustrated in **Figure 1**. The gills are covered by a squamous, non-keratinized epithelium that, in zebrafish, is mainly composed of pavement cells, ionocytes (mitochrondria-rich cells α and β), mucous cells and neuroepithelial cells. The entire surface of the gills constitutes a large and complex mucosal territory, with many microenvironments, that separates the external environment from the blood flow. As such, the gill mucosa clearly represents a key potential gateway for pathogens to enter the host (52).

The existence of a gill-associated acquired immunity in fish was first reported in 1919 (53). Xu and colleagues showed in trout that gill infections with the parasite *Ichthyophthirius multifiliis* induce a strong local pathogen-specific IgT (fish mucosal immunoglobulin, named IgZ in zebrafish) response (54). IgM and IgT antibody responses seem to be a conserved defense mechanism within fish MALTs, although not all fish species possess IgT (55). However, much remains unknown about the process of gill immunity. The maintenance of such a delicate respiratory barrier would likely require finely-tuned regulatory safeguards. Indeed, it is important to avoid unnecessary, or exagerated, immune responses against non-harmful foreign elements, such as commensal bacteria and innocuous environmental debris. The anti-inflammatory cytokine IL-10 seems to be an important factor for gill homeostasis in zebrafish (56). Zebrafish gill’s defenses consist of many scattered myeloid cells, lymphoid cells, and myeloid-like metaphocytes (16, 21, 44, 57). The sampling of foreign antigens is performed by both epithelial cells and immune cells from the gill mucosa. The presence of specialized antigen-sampling cells at the surface of the gill epithelium has been reported in trout but not zebrafish (58). We recently reported the presence of an ILT in zebrafish gills that is structurally similar to the ILT of salmonids (44). The zebrafish ILT forms a T-lymphocyte-rich region on top of the interbranchial septum, called the “proximal” ILT (pILT). The ILT also extends along the afferent aspect of each gill filament, forming a “distal” ILT (dILT) (59). In addition to the zebrafish ILT, our recent study also shows the presence of unidentified lymphoid aggregates in gills of several neoteleost species, suggesting that our knowledge of the fish GIALT organization remains incomplete.

Here, we explored the structural organization of the adult zebrafish GIALT (zf-GIALT) with an immuno-histology approach coupled to 3-D, high-resolution microscopy. Using a monoclonal antibody targeting a highly conserved epitope of the kinase ZAP70 (44), we labeled T/NK-like cells on cryosections of non-dissected gills and produced images at high-magnification and high-resolution. With this approach, we could separate the zf-GIALT into different sub-regions, including ILT and a so far undescribed structured lymphoid tissue located along each side of the gill arches at the base of filaments. Here, we term this structure as the “Amphibranchial Lymphoid Tissue” (ALT). Our results show that the ILT and the ALT share features at the structural and cellular levels, and show that both are subject to structural changes upon infection by the Spring Viraemia of Carp Virus (SVCV), suggesting an involvement in acquired immunity. Finally, ALT was found in all teleost species we investigated, even in those species previously shown to lack ILT, emphasizing that it may constitute a primordial and conserved lymphoid structure involved in the protection of teleost gills.

## Material and Methods

### Animal Care and Ethic Statement

Experiments were performed in accordance with the animal care guidelines, ethical standards and legislation of the European Union, France and Norway. The study was carried in contact with local ethics committees. Animal experiments described in the present study and conducted at the IERP fish facilities (building agreement n°C78-720, doi.org/10.15454/1.5572427140471238E12) of the INRAE Research Center at Jouy-en-Josas, France, in compliance with the recommendations of Directive 2010-63-EU on the protection of animals used for scientific purposes. Infection protocols were approved by the institutional review ethics committee, COMETHEA, of the INRAE Research Center. Authorizations were approved by the French Ministry of Agriculture (authorization number APAFIS# 22150-2019062811528052).

This study was performed by well-trained staff authorized to perform fish euthanasia.

The experiments were conducted using wild-type AB zebrafish (1 year old) (n = 31) and the following genetically-modified strains: *Tg(mhc2dab:GFP)^sd6^* (15) (n=3), *Tg(mfap4:mCherry-F)^ump6^* (60) (n=3), *Tg(mpx:GFP)^i114^* (61) (n=3), *Tg(rag2:DsRED)* (62) (n=3) and *Tg(fli1a:EGFP)^y1^* (63) (n=3). In addition, laboratory-raised wild-type d-rR adult Medaka (1 year old) (n=3), that were maintained in the conditions presented in (64), were provided by Romain Fontaine (NMBU – Ås, NORWAY).

Healthy adult Atlantic salmons (n=2, weight: 1500g) were laboratory-raised by NIVA (Solbergstrand-NORWAY) and provided by PHARMAQ, part of Zoetis. Euthanasia of the specimens followed strictly the Norwegian legislation and was carried out by an authorized staff.

Healthy common carp (*Cyprinus carpio*) (n=32, weight: 300-2000g) were raised in tanks of recirculating, UV-treated water (23°C ±1°C, 12:12 light: dark photoperiod) at the aquatic research facility of Wageningen University & Research, the Netherlands. No disease had been observed in the fish in the recent past.

The study includes wild-caught species for which only fish with a healthy appearance upon sampling and no obvious histopathological changes in gill tissue upon histological evaluation were included in the study. This included Atlantic mackerel (*Scomber scombrus*) (n=5, weight: 465-910g, from Drøbakksundet, Fjord of Oslo, Norway), Northern red snapper (*Lutjanus campechanus*) (n=3, weight: 600-850g, from Turks and Caicos Islands), and Pacific barracuda (*Sphyraena argentea*) (n=3, weight: 500-800g, Turks and Caicos Islands).

### Infection Experiment With SVCV

Wild-type zebrafish (AB; aged 15 months; 0.8g) were acclimatized at 22°C (Ph 8, conductivity 200µS/cm²) in 1.5L aquaria for 48h. Two groups of eight fish were infected by immersion using the reference SVCV strain VR-1390 (65, 66) at 10^4^ pfu/ml and the water flow was stopped for 48 h; water was then changed once per day. Infected groups were sampled at 3 and 10 days post infection, respectively and fixed with a 4% formaldehyde solution. Non-infected controls were prepared in parallel (n = 4).

### Sample Preparation and Sectioning

#### Cryosections

Adult zebrafish and medaka were euthanized with an anesthetic overdose using buffered tricaine. The specimens were then quickly transferred to a solution of formaldehyde 4% in HEPES buffer (60 mM – pH 7.4) for 24 h at room temperature, followed by a 3 days incubation at 4°C. The cryoprotection was achieved with two incubations in a solution of sucrose- 32% (in distilled water), until the specimens sunk to the bottom of the recipient. The samples were then embedded in Tissue-Tek O.C.T. Compound (Sakura Finetek USA, Mountain View, CA, USA), flash-frozen in isopentane, and sectioned using a CM1950 cryostat (Leica, Wetzlar, Germany). The resulting 30 µm cryosections were recovered on Superfrost Plus slides (Thermo Fischer, Waltham, MA, USA) and stored at -20°C.

#### Wholemount 

Adult zebrafish were euthanized with an anesthetic overdose using buffered tricaine. The specimens were then quickly transferred to a solution of formaldehyde 4% in HEPES buffer (60 mM – pH 7.4) for 24 h at room temperature, followed by a 3 days incubation at 4°C. Gill arches were gently micro-dissected and stored in HEPES buffer (60 mM – pH 7.4) at 4°C.

#### Paraffin Sections

Samples from the relevant species where prepared as described in (44). Briefly, tissues were fixed in 10% neutral buffered formalin and stored at 4°C before further histological processing. Upon processing, the tissues were dehydrated, cleared, paraffin-embedded and processed using standard procedures. Tissues were then sectioned at 4 µm, then were recovered on poly-L-lysine coated Superfrost Plus slides (Thermofisher, Waltham, MA, USA) and stored at 4°C.

### Immunofluorescence and Immunohistochemistry

#### Cryosections

Slides were blocked 1h in a blockaid solution (Thermofisher) at room temperature. T/NK lymphocytes were labeled using a rabbit anti-ZAP70 monoclonal antibody (99F2 – Cell Signaling), at 1:300 in Pierce™ Immunostain Enhancer solution (Thermofisher) for 1 h 40 min at room temperature. Cytokeratin was labeled using the cytokeratin Pan Type I/II mouse monoclonal antibody cocktail (Thermofisher), at 1:40 in PHEM buffer (60 mM PIPES, 25 mM HEPES, 10 mM EGTA and 20 mM MgCl_2_ in ddH_2_O – pH 7.4) (67, 68) for 1 h at room temperature. Dendritic cells were labeled using peanut agglutinin lectin coupled with alexa594 (Thermofisher), at 1:200 in PHEM buffer for 25 min at room temperature. T/NK cell proliferation was assessed by first labeling proliferating cells with a mouse anti-PCNA monoclonal antibody (PC10 – Thermofisher) at 1:300 in PHEM buffer for 1 h 40 min at room temperature, followed by an anti-ZAP70 labeling as described above. SVCV was revealed using a mouse anti-SVCV-N monoclonal antibody (BIO 331 – Bio-X Diagnostics) at 1:20 in PHEM buffer for 1 h at room temperature, alone, followed by anti-ZAP70 labeling as described above, or along with a rabbit polyclonal anti-MPEG1 antibody (Batch RC1201 - Anaspec) at 1:250. When necessary, the labeling was followed by a 30 min incubation at room temperature with one or several cross-adsorbed secondary antibodies at 1:250 in PHEM buffer: Goat anti-rabbit-Alexa647 (Jackson ImmunoResearch), Goat anti-mouse-Alexa647+ (Thermofisher) and Goat anti-mouse-Dylight594 (Thermofisher). Where relevant, slides were co-stained with fluorescent phalloidin (TRITC or FITC labeled – Sigma Aldrich) at 3U/mL, and DAPI (Thermofisher) at 5 µg/mL during the incubation with secondary antibodies or peanut agglutinin lectin. Slides were mounted with prolong-glass mounting medium (Thermofisher), cured at room temperature for 24 h and stored at 4°C before confocal imaging. Rabbit IgG isotype controls (DA1E – Cell Signaling) for the anti-ZAP70 labeling in the ILT and ALT are displayed on **Figure S1**.

#### Wholemount

Dissected gill arches were permeabilized for 5 days in a solution of 1% triton X-100 (Sigma Aldrich) and 0,1% tween (Sigma Aldrich) in PHEM buffer for 5 days at room temperature under gentle rocking. Samples were then saturated, under gentle rocking, for 24 h at room temperature in a solution of blockaid complemented with 0,5% triton X-100 and 0,1% tween. T/NK lymphocyte were labeled with the rabbit anti-ZAP70 monoclonal antibody at 1:600 in Pierce™ Immunostain Enhancer solution, complemented with 0,5% triton and 0,1% tween, for 5 days at room temperature. After 24 h with several rinses in 0,5% triton and 0,1% tween in PHEM buffer, the samples were incubated with a mix of Goat anti-rabbit-Alexa647 at 1:400, phalloidin-FITC at 2U/mL and DAPI at 5 µg/mL, for 5 days at room temperature under gentle rocking. After several rinses, the samples were mounted with slowfade-glass mounting medium (Thermofisher) between two coverslips with adequate spacers, and imaged during the following hour.

#### Paraffin Sections

A protocol adapted from Dalum et al. (69) was employed on formalin-fixed, paraffin-embedded gill tissues attached to poly-L-lysine coated Superfrost Plus slide. In short, prepared slide were submitted to deparaffinization, heat-induced epitope retrieval, inhibition, and blocking, before the slides were incubated with a primary antibody, the anti-ZAP70 monoclonal antibody as described above, at 1:300 in 1% bovine serum albumin/tris-buffered saline. As secondary antibody, labelled polymer-HRP anti-rabbit (Dako EnVison1 System-HRP, Dako, Glostrup, Denmark) was used, followed with incubation with 3,30-diaminobenzidine (DAB) as substrate (positive reaction indicated by brown color). Pre-immune sera and omissions of primary antibodies were included as a negative control. Counterstaining was performed with haematoxylin, followed by washing in distilled water and mounting with polyvinyl alcohol (Apotekproduksjon, Oslo, Norway).

### Imaging and Image Analysis

Acquisitions were made with the Zyla camera of a dragonfly 500 spinning disk confocal microscope (Andor, Belfast, UK), with 40 µm pinholes and either a 20×/0.75-dry objective or a 60×/1.4-oil-immersion objective. Acquisitions, stitches and deconvolutions (14-16 iterations) were made using features from the Fusion software. Image analysis was realized using both IMARIS and ImageJ softwares. Image acquisition and analysis were performed at the NorMIC imaging platform (University of Oslo, Norway).

## Results

### General Organization of the zf-GIALT

**Figure 2:**
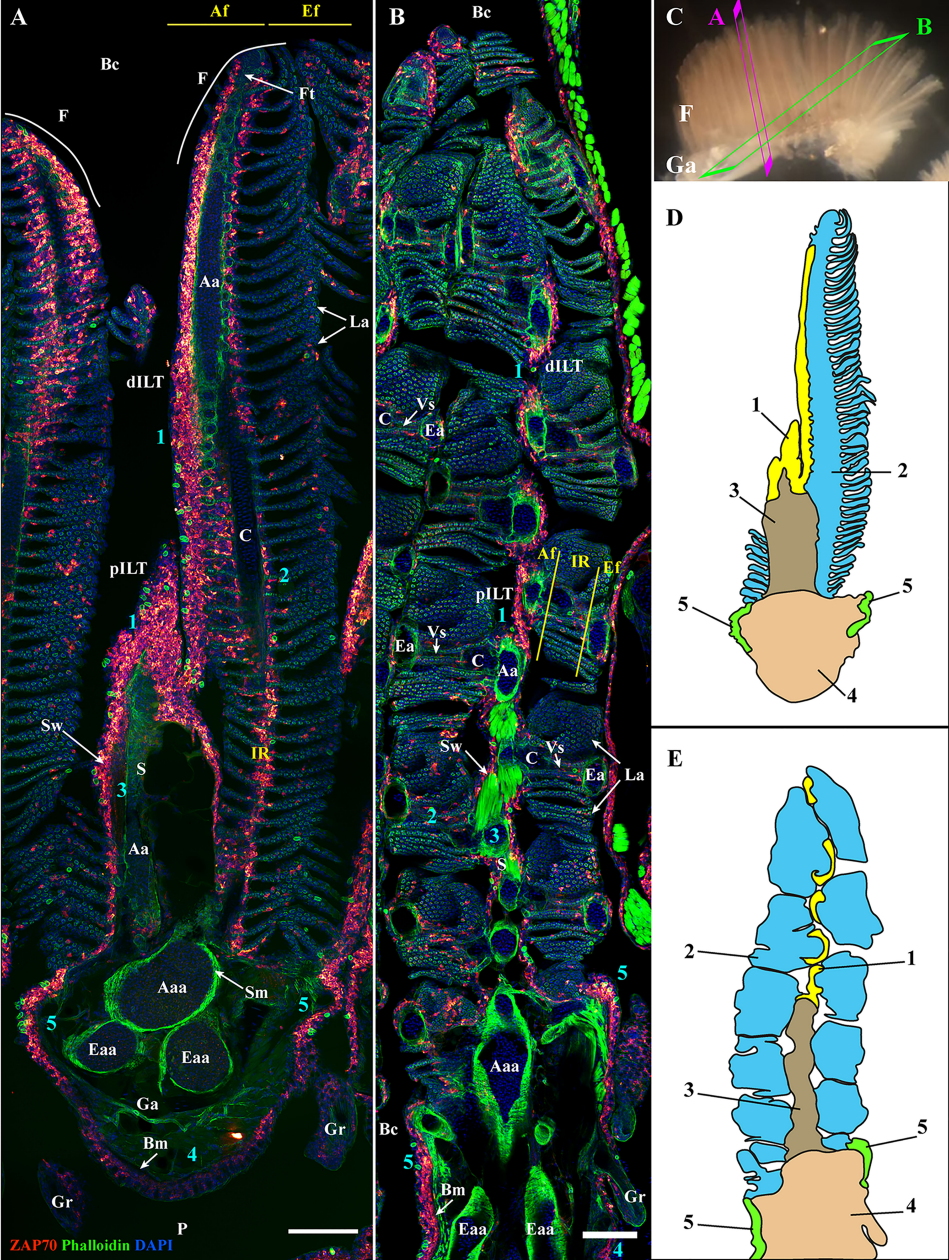
General organization of the zebrafish GIALT. Representative deconvolved confocal images of adult zebrafish gills acquired from a transversal **(A)** and oblique longitudinal orientations **(B)**. The section planes are illustrated on a dissected gill arch in **(C)**. Images were acquired from 30 μm whole-body cryosections stained with phalloidin (actin -green) and DAPI (DNA-blue) and where T/NK cells were labeled with anti-ZAP70 antibody (red hot). Both transversally and longitudinally sectioned gills display a distribution of ZAP70 positive cells that is connected to the different morphological territories of the gills, thus revealing the segmentation of the GIALT into five sub-regions (1-5) (ILT, interlamellar region-lamellae-efferent aspect of filaments, interbranchial septum, gill arch, T/NK cell clusters at the base of filaments on each side of the gill arch). **(D, E)** Schematic representation of **(A, B)** displaying the 5 sub-regions of the GIALT. **(A, B)** Images are maximum intensity projections (MIP). Annotations: Aa, Afferent artery; Aaa, Afferent arch artery; Af, Afferent aspect of filaments; Bc, Branchial cavity; Bm, Basement membrane; C, Cartilage; dILT, distal Interbranchial Lymphoid Tissue; Eaa, Efferent arch artery; Ea, Efferent artery; Ef, Efferent aspect of filaments; F, Filament; Ft, Filament top; Ga, Gill arch; Gr, Gill raker; IR, Interlamellar region; La, Lamellae; P, Pharynx; pILT, proximal Interbranchial Lymphoid Tissue; S, Septum; Sm, Smooth muscles; Sw, Septum wall and Vs, Venous sinus. Scale bars: 100 μm **(A, B)**.

Since gills have a very complex architecture, we first explored the distribution of the anti-ZAP70 labeling on 3D multi-fields of view acquisitions of gills sectioned at multiple orientations: transversal and oblique coronal (**Figure 2**) (n = 5). These initial observations revealed abundant ZAP70+ T/NK-like cells distributed throughout the gills. However, their distribution was not uniform. Based on the morphological compartmentalization of the gills (**Figure 1**) and the distribution pattern of the anti-ZAP70 labeling, the zf-GIALT could be divided into five sub-regions. The first sub-region (1) constitutes a T/NK cell-rich structure located on top of the septum that extends along the afferent aspect of the filament. This is the ILT, as recently described in (44). The second sub-region (2) consists of scattered T/NK cells within the interlamellar region, the efferent aspect of the filament, and the lamellae. The third sub-region (3) contains scattered T/NK cells that are found along the wall of, and within, the septum. The fourth sub-region (4) relates to scattered T/NK cells at the pharyngeal side and within the gill arch. Importantly, a fifth sub-region (5) could be identified by a high number of T/NK cells clustered at the base of the efferent aspect of the filaments, on each side of the gill arch. This last sub-region was surprisingly large and resembled the lymphoid aggregates we previously observed at the base of the filament of gills from spiny-rayed fish species: the European perch (*Perca fluviatilis*) and the Flounder (*Glyptocephalus cynoglossus*) (44).

**Figure 3:**
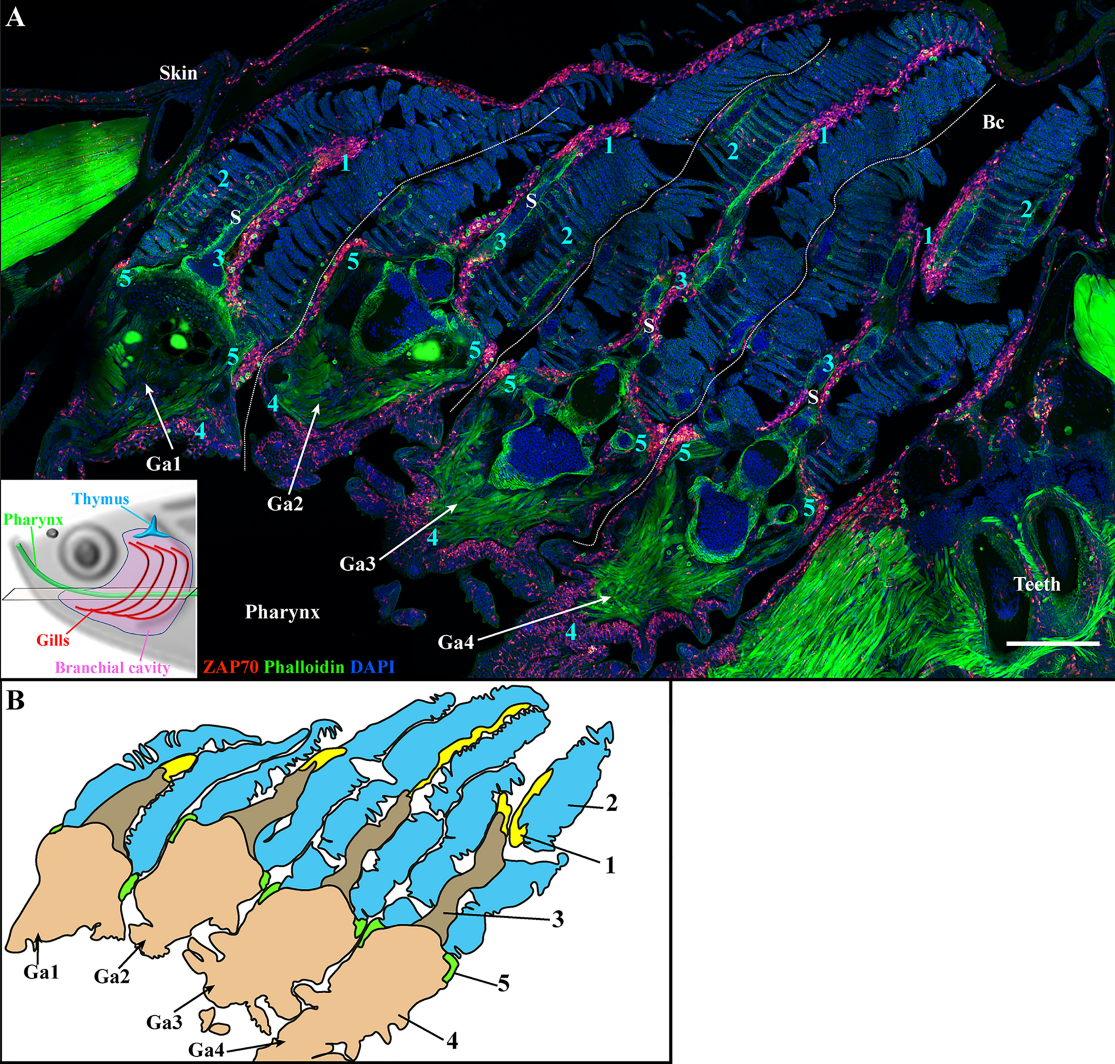
The four zebrafish gill arches display the same lymphoid organization. Representative deconvolved confocal images of an adult zebrafish branchial cavity showing the 4 gills arches with transversal orientation **(A)**. The plane of section is illustrated by the scheme at the bottom left. The images were acquired from 30 μm whole-body cryosections stained with phalloidin (green) and DAPI (blue) and where T/NK cells were labeled with anti-ZAP70 antibody (red hot). Each gill arch (Ga1-Ga4) possesses a GIALT segmented in five sub-regions (1-5) (ILT, interlamellar region-lamellae-efferent aspect of filaments, interbranchial septum, gill arch, T/NK cell clusters at the base of filaments on each side of the gill arch). **(B)** Schematic representation of **(A)** displaying the 5 sub-regions of the GIALT. **(A)** The image is a maximum intensity projection of 15 µm. Annotations: Bc, Branchial cavity; Ga, Gill arch and S, Septum. Scale bar: 200 μm.

The zebrafish has four pairs of gill arches that display slight morphological differences between them. Gill rakers from the first gill arch are most developed, which restrains the passage of larger food particles into the gills. To verify that the organization of the zf-GIALT reported above is similar in all four pairs of gill arches, we made coronal cryosections through the head of adult zebrafish such that, if when one looked at a branchial cavity, the four gill arches could be observed in a transversal orientation. As illustrated in **Figure 3**, these sections clearly confirm that all gill arches share the same lymphoid organization (n = 3). Furthermore, this organization of the zf-GIALT was confirmed in all analyzed specimens (**Figure S2**). Dynamic visualizations of the different sub-regions are available as **Supplementary Videos 1–4**.

Based on these observations, we next performed a more detailed characterization of the five different sub-regions of the zf-GIALT (1-5), using high-resolution 3D imaging.

### The Interbranchial Lymphoid Tissue (ILT) – Sub-Region 1

**Figure 4:**
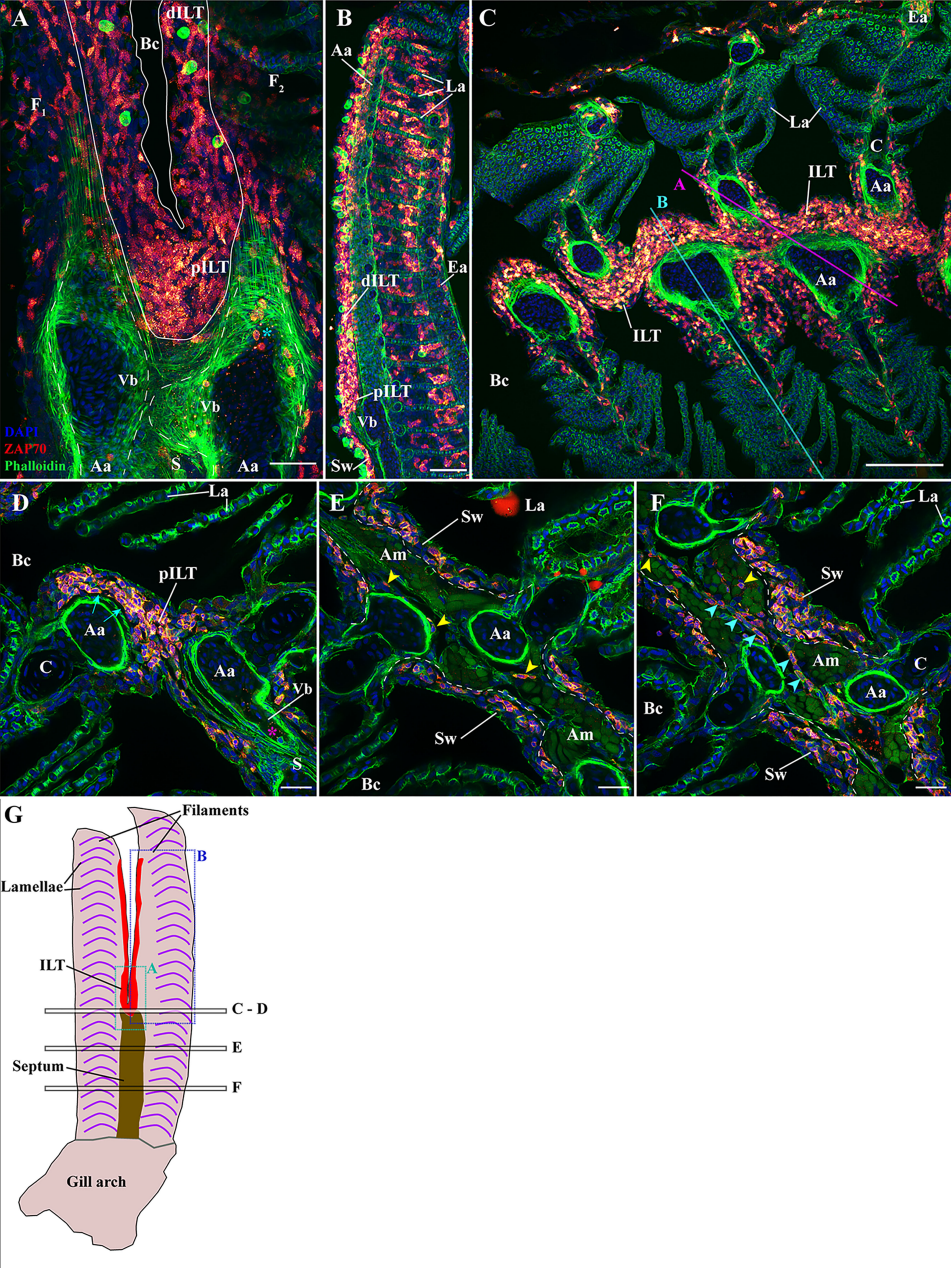
Organization of the zf-GIALT above the gill arch. (Sub-regions 1-3). Representative deconvolved confocal high-resolution images of adult zebrafish gills acquired with a transversal **(A, B)** or coronal orientations **(C–F)**. The different section planes are illustrated on panels **(C)** and in **(G)**. Images were acquired from 30 μm whole-body cryosections stained with phalloidin (green) and DAPI (blue) and where T/NK cells were labeled with anti-ZAP70 antibody (red hot). **(A)** An overview of the top of an interbranchial septum showing the location of the proximal ILT over the vascular bleb from two opposite filaments. The cyan star indicates T/NK cells adhering to the endothelium of the vascular bleb. **(B)** Transversal acquisition through the middle of a filament illustrating the heterogeneity of the distribution of ZAP70 positive cells within a filament. **(C)** Coronal section across the proximal ILT showing the continuity of the ILT across the branchial cleft. **(D–F)** High-resolution images acquired with coronal orientations at different levels through the filaments [see panel **(G)**] and illustrating the zf-GIALT associated to the interbranchial septum. The cyan arrows in **(D)** indicate the basement membrane separating the vascular compartment from the ILT, the magenta star highlights a ZAP70_neg_ immune cells within the vascular bleb, yellow arrowheads in **(E, F)** point to scattered T/NK cells within the septum, and cyan arrowheads in **(F)** highlight T/NK cells within a vessel of the septum. Images are maximum intensity projections: 2 µm **(D–F)**. Annotations: Aa, Afferent artery; Am, adductor muscles; Bc, Branchial cavity; C, Cartilage; dILT, distal Interbranchial Lymphoid Tissue; Ea, Efferent artery; F, Filament; La, Lamellae; pILT, proximal Interbranchial Lymphoid Tissue; S, Septum; Sw, Septum wall and Vb, Vascular bleb. Scale bars: 100 μm **(C)** 40 μm **(B)** 30 μm **(A)** and 20 μm **(D–F)**.

The morphology of the Interbranchial lymphoid tissue in adult zebrafish is very similar to the one observed in Atlantic salmon. Rich in T/NK cells, the tissue is located intraepithelial on the apex of the septum, where it is called the proximal ILT (pILT), and along the afferent aspect of filaments where it constitutes the distal ILT (dILT) (44) (**Figures 4A, B**). The ILT is restricted to the afferent aspect of filaments, at the surroundings of afferent arteries and filament cartilages. *Via* its tight association with the septum, the ILT is continuous throughout the length of the epithelium covering the branchial cleft (**Figure 4C**). A peculiarity of this association with the septum is that the ILT lies above an enlargement of the afferent artery that forms closed pits on the radial axis (**Supplementary Videos 5, 6**), called “Vascular bleb” or “Ampulla” (Vb in **Figures 4A, B**) (70). Finally, the ILT is separated from the vascular compartment by a thin basement membrane that appears as a black line in our acquisitions (**Figure 4D** – cyan arrows). These two last features may promote exchanges between the ILT and the vascular system, and surrounding tissues, possibly facilitating the maintenance of homeostasis and immune function.

### The zf-GIALT at the Lamellae, Filament, and Interbranchial Septum – Sub-Regions 2-3

Outside the ILT, a few T/NK cells were found scattered in the different layers of the filaments. Occasional T/NK cells were also observed within the thin respiratory epithelium of the lamellae, but very few were seen within the blood circulating between the pillar cells (ring-like structures labelled by phalloidin (actin) staining). At the interlamellar region, some T/NK cells were observed around the central venous sinus and within the epithelium (**Figure 4C**), a site where they sporadically form aggregates but not organized structures (**Figures 2A**, **4B**). The distribution of the T/NK cells at the efferent aspect of the filament follows a similar pattern as the scattered T/NK cells found along the epithelium, which can form a few localized aggregates.

We then studied the organization of the zf-GIALT at the interbranchial septum. Here, a high number of T/NK cells were consistently found within the wall of the septum (**Figures 2** **–** **4**). Inside the deeper layers of the septum, T/NK cells were scattered between muscle cells (**Figures 4E, F** – yellow arrowheads) and in association to narrow endothelial structures revealed by the phalloidin staining for actin (**Figure 4F** – cyan arrowheads).

### The zf-GIALT at the Gill Arch – Sub-Region 4

**Figure 5:**
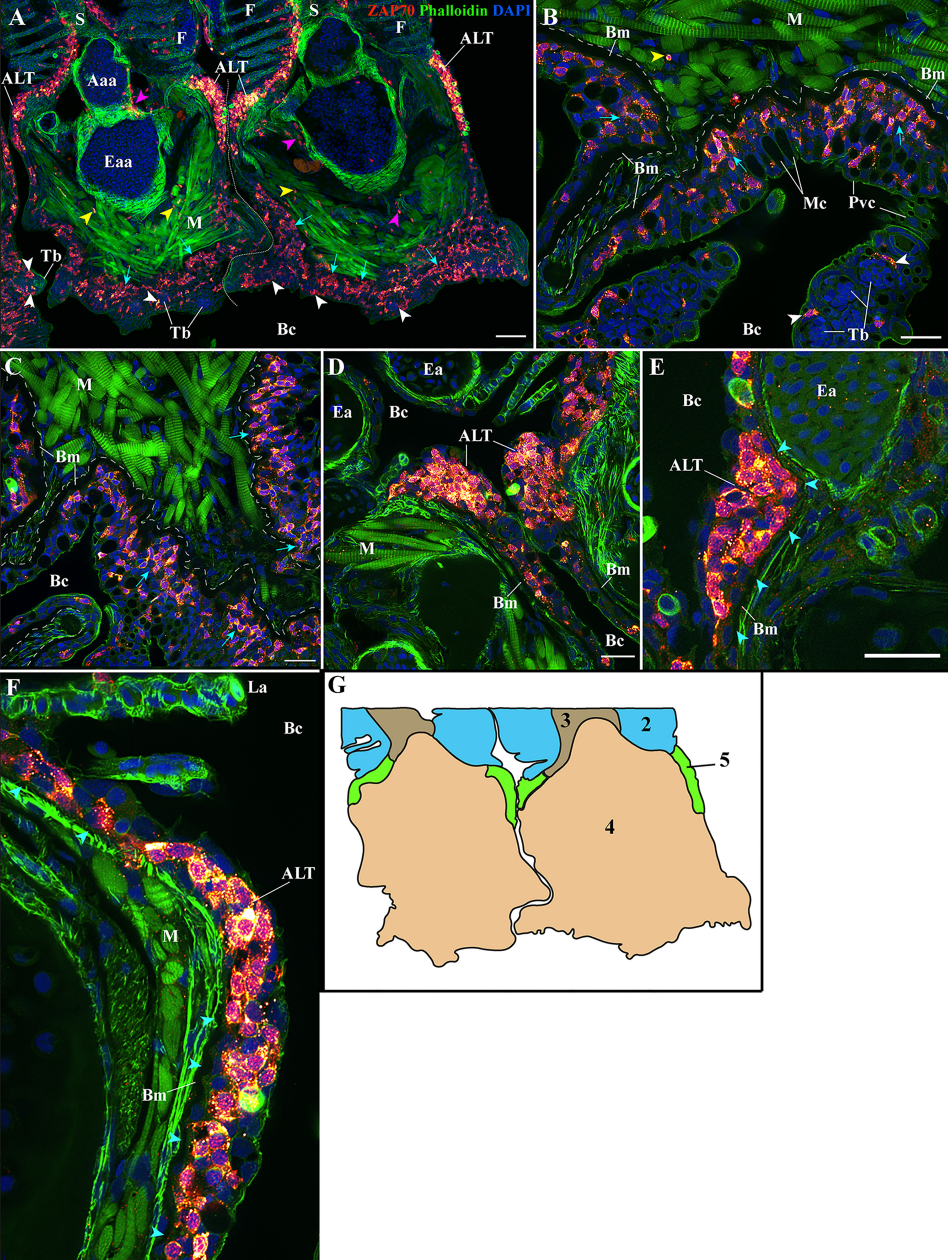
Organization of the zf-GIALT at the gill arch. (Sub-regions 4-5). Representative deconvolved confocal high-resolution images of adult zebrafish gills acquired with a transversal orientation. Images were acquired from 30 μm whole-body cryosections stained with phalloidin (green) and DAPI (blue) and where T/NK cells were labeled with anti-ZAP70 antibody (red hot). The localization of the GIALT sub-regions is illustrated in **(G)**. **(A)** Lower magnification image illustrating the organization of the GIALT from two gill arches. Cyan arrows point to the numerous T/NK cells close to the basement membrane at the pharyngeal side of the gill arch. Yellow arrowheads highlight scattered T/NK cells within the gill arch, and white arrowheads localize taste buds. **(B, C)** Images at higher magnification of the zf-GIALT at the pharyngeal aspect of the gill arch. **(D–F)** Images at higher magnification of the T/NK cell clusters, forming the Amphibranchial Lymphoid Tissue (ALT), located on each side of the gill arches, at the base of filaments. The cyan arrowheads in **(E, F)** highlight the basement membrane and the reduction of its thickness at the level of the ALT. Images are maximum intensity projections: 2 µm **(B–F)**. Annotations: Aaa, Afferent arch artery; ALT, Amphibranchial Lymphoid Tissue; Bc, Branchial cavity; Bm, Basement membrane; Eaa, Efferent arch artery; Ea, Efferent artery; F, Filament; La, Lamellae; M, Muscles; Mc, Mucous cell; Pvc, Pavement cell; S, Septum and Tb, Taste bud. Scale bars: 50 μm **(A)** and 20 μm **(B–F)**.

In the gill arch, *i.e.* in the area beneath the filaments and septum, we observed a few scattered T/NK cells between muscle cells (**Figures 5A, B** yellow arrowheads), sometimes associated with the circulatory system (**Figures 5A, B** magenta arrowheads). In contrast, the epithelium on the pharyngeal side was populated by many T/NK cells, in a disorganized fashion. Still, we noticed that these intra-epithelial T/NK cells were preferentially located in the basal layers of the epithelium (**Figures 5A–C** cyan arrows), in proximity to a thick basement membrane, hinting toward a potential involvement of the gill arch epithelial basement membrane in T/NK cell trafficking. Noteworthy, some T/NK cells were also found in association with taste buds (**Figures 5A–C** white arrowheads).

### Identification of the Amphibranchial Lymphoid Tissue (ALT) – Sub-Region 5

The presence of large clusters of T/NK cells on each side of gill arches, at the base of the efferent aspect of filaments, was unexpected. Our investigations revealed that these clusters constitute a new structured lymphoid tissue, which we name the “Amphibranchial Lymphoid Tissue” (ALT). The rationale behind the identification and the description of the ALT are exposed in the following paragraphs.

These dense T/NK cells aggregates were in sharp contrast to the rest of the gill arch epithelium, which had numerous but always scattered T/NK cells (**Figure 5A**). Within these clusters, the T/NK cells were in contact with each other (**Figures 5D–F**) and formed a well-delineated bulge in the epithelium. From section to section, the thickness of these lymphoid clusters was variable and sometimes appeared narrow (**Figure 5E**). Intriguingly, these clusters also coincided with a reduction in the thickness of the epithelial basement membrane, to the point that it was sometimes difficult to follow its continuity (**Figures 5E–F** – cyan arrowheads).

**Figure 6:**
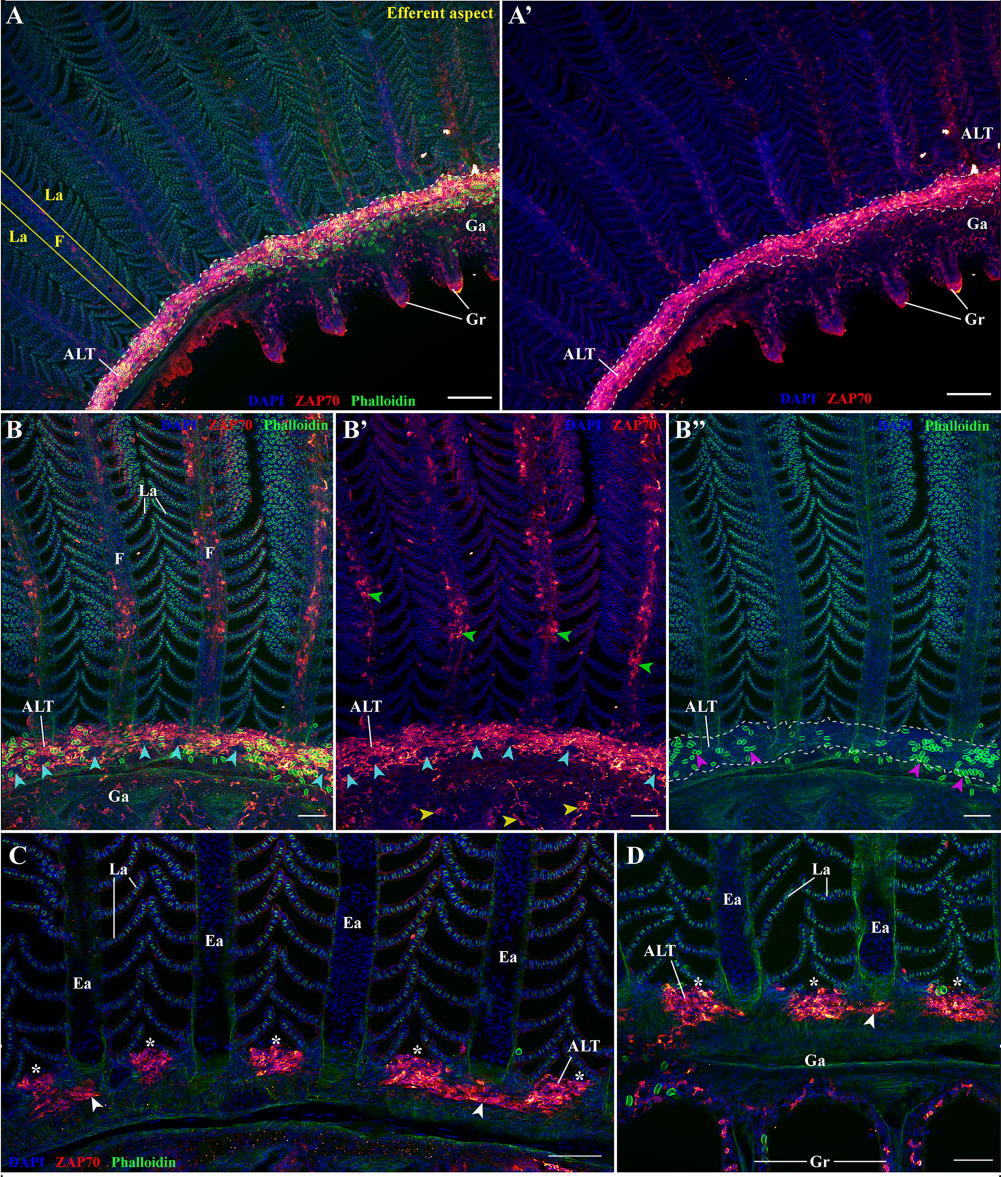
The amphibranchial lymphoid tissue is a continuous structure along gill arches. Representative deconvolved confocal images of an adult zebrafish gill arch observed from the side. The images were acquired from wholemount dissected gill arches stained with phalloidin (green) and DAPI (blue) and where T/NK cells were labeled with anti-ZAP70 antibody (red hot). **(A,A’)** low magnification image of the gill arch illustrating the continuity of the ALT. **(B–B’’)** Images of the ALT at a higher magnification showing the contrast between the lymphoid organization of the ALT, the efferent aspect of filaments (green arrowheads), and the pharyngeal side of the gill arch (yellow arrowheads). Magenta arrowheads in **(B’’)** emphasize the enrichment of the ALT in rodlet cells, while the cyan arrowheads in **(B, B’)** point to the localization of neuromast-like ZAP70 negative structures. **(C, D)** Optical sections from within 3D acquisitions illustrating the variating thickness of the ALT. The white arrowheads point to the thinness of the ALT when at the level of the efferent artery compared to the thickness of the ALT when in-between filaments. **(D)** is deeper within the 3D acquisition than **(C)**. **(A–D)** Images are maximum intensity projections: 5 μm **(C, D)**, 25 μm **(B–B’’)** and 50 μm **(A, A’)**. Annotations: ALT, Amphibranchial Lymphoid Tissue; Ea, Efferent artery; F, Filament; Ga, Gill arch; Gr, Gill raker and La, Lamellae. Scale bar: 20 μm **(D)**, 40 μm **(B, C)** and 100 μm **(A, A’)**.

Since these clusters were consistently seen in all transversal sections we analyzed, we wondered if they were localized aggregates or if these structures were continuous through the length of the gill arch. To answer this question, we performed ZAP70 labeling on wholemount fixed dissected gill arches that were then imaged with a sagittal orientation (**Figure 6** and **Supplementary Video S7**) (n = 3). The maximum intensity projections clearly revealed that these clustered T/NK cells were more than just localized lymphoid aggregates but actually formed a continuous structure through the whole length of the gill arch, at the base of the filaments (**Figures 6A, B’’**). The acquisition also further illustrates the scattered nature of the GIALT at the efferent aspect of filaments (green arrowheads) and at the gill arch (yellow arrowheads). Curiously, we could also observe an enrichment in rodlet cells (magenta arrowheads) and the presence of ZAP70-negative round structures within this lymphoid structure (cyan arrowheads) (**Figure 6B–B”**). Further examinations revealed these ZAP70 negative structures to be neuromast-like or taste-bud like structures (**Figure S3** and **Supplementary Video S8**). To the best of our knowledge, these likely sensory structures have until now not been described and may represent a neuro-immune unit in teleosts.

Opening-up the acquired 3D stacks to analyze the optical sections revealed that this ZAP70-enriched structure consists of prominent and dense aggregates in-between the filaments, (white stars) and is at its thinnest at the level of the efferent artery (white arrowheads) (**Figures 6C, D**).

**Figure 7:**
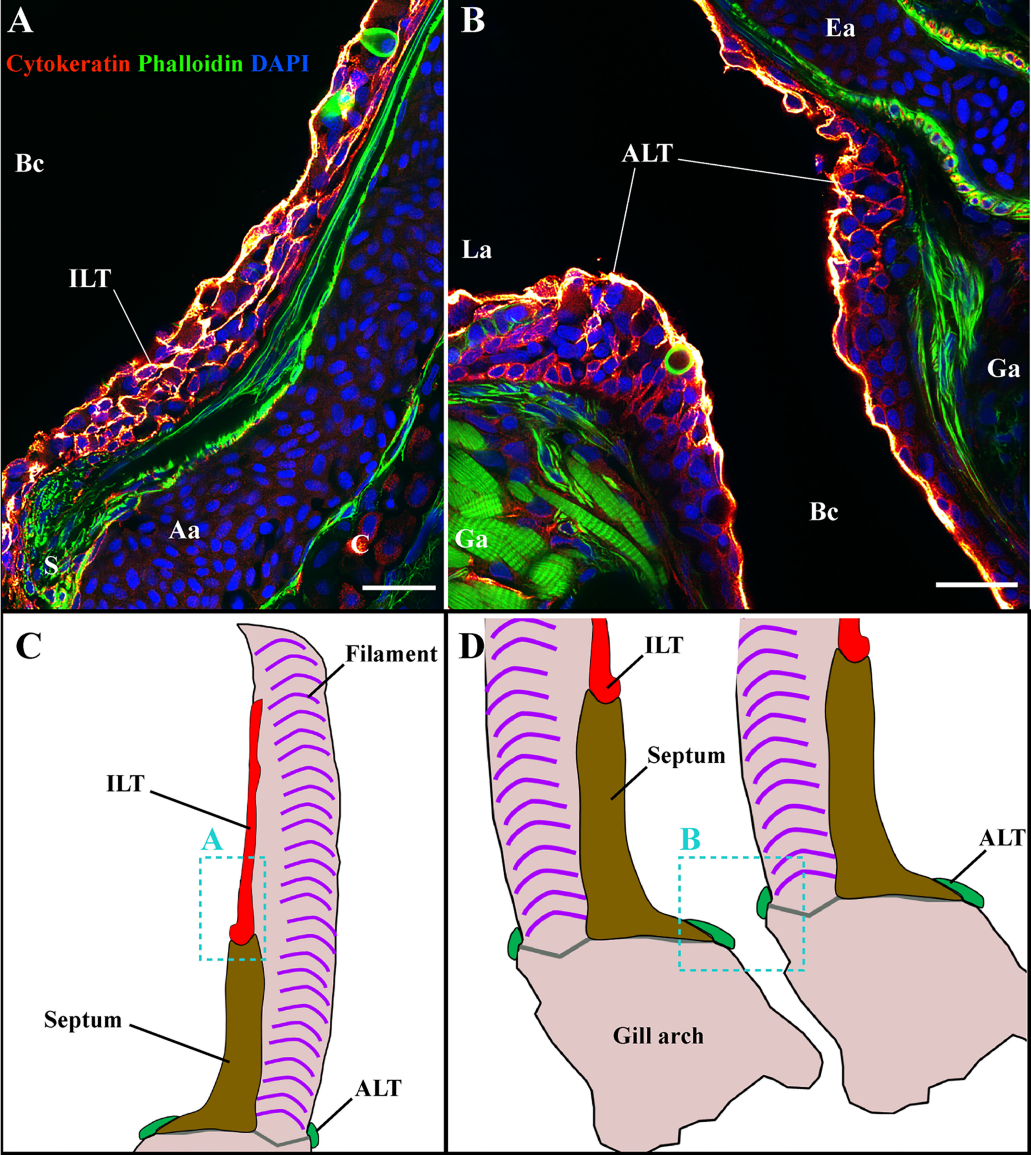
The ILT and ALT are structured by a complex network of reticulated epithelial cells. Representative deconvolved confocal images of adult zebrafish gills displaying the ILT **(A)** and the ALT **(B)** at high-magnification. The localization of sections **(A, B)** is illustrated on panels **(C, D).** The images were acquired from 30 μm whole-body cryosections stained with phalloidin (green) and DAPI (blue) and reticulated epithelial cells were labeled with an anti-cytokeratin antibody (red hot). While the cytokeratin signal is strong within the outermost layer of the epithelium, an intricate core of cytokeratin positive cells is observed within ILT **(A)** and ALT **(B)**. Images are maximum intensity projections (2 µm). Annotations: Aa, Afferent artery; ALT, Amphibranchial Lymphoid Tissue; Bc, Branchial cavity; C, Cartilage; Ea, Efferent artery; Ga, Gill arch; ILT, Interbranchial Lymphoid Tissue; La, Lamella and S, Septum. Scale bars: 20 μm.

**Figure 8:**
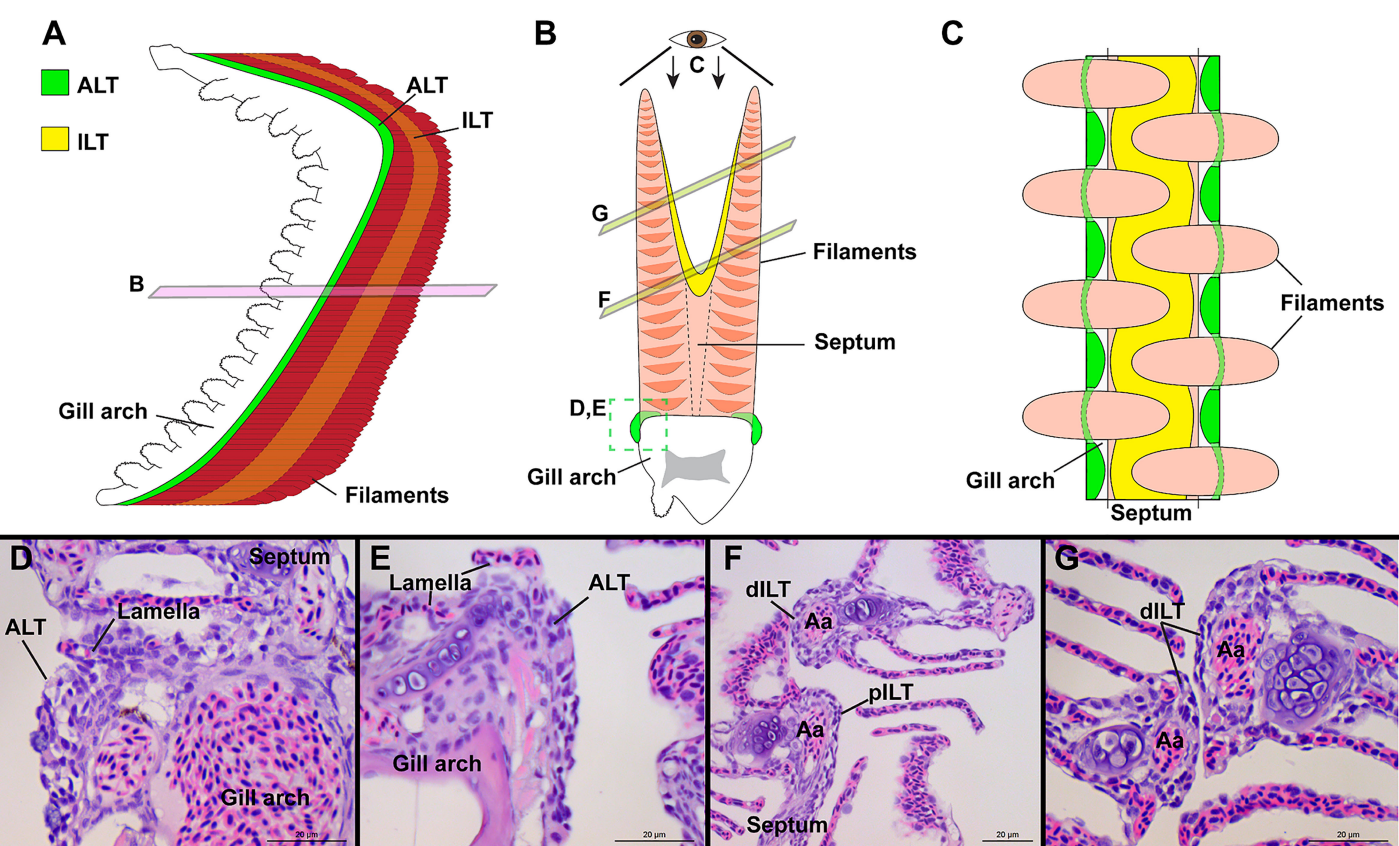
General organization of the structure lymphoid tissues in zebrafish gills. Schematic representations of the localization of the ALT (green) and ILT (yellow) within adult zebrafish gills. The ALT runs along the base of filaments while the ILT is found on the inner (afferent) aspect of filaments, on top of the interbranchial septum. **(A)** illustrates a gill arch as observed from the side. **(B)** represents a transversal plane through the gill arch, as illustrated on **(A)**. **(C)** shows a gill arch as observed from above the filaments, as illustrated in **(B)**. **(D, E)** Paraffin sections (2 µm) displaying the ALT, as illustrated in **(B)**. **(F, G)** Paraffin section (2 µm) displaying the proximal ILT (pILT) and distal ILT (dILT), as illustrated in **(B)**. Aa, Afferent artery; ALT, Amphibranchial Lymphoid Tissue; ILT, Interbranchial Lymphoid Tissue; dILT, distal Interbranchial Lymphoid Tissue and pILT, proximal Interbranchial Lymphoid Tissue. Scale bars: 20 µm **(D–G)**.

Combined, our results indicate that the T/NK cell organization found in this sub-region 5 is strikingly similar to what is seen in the ILT. With such an arrangement, an important question arose: Are these structures along the sides of gill arches only diffuse lymphoid aggregates, or could they represent a new structured gill intra-epithelial lymphoid tissue? To address this question, we looked for an important feature of structured lymphoid tissues, the presence of a reticulated epithelial cells network. For this, we labeled cryosections with an anti-cytokeratin antibody to reveal the reticulated epithelial cells (**Figure 7**) (n = 3) as previously described by Haugarvoll et al. in their identification of the ILT in Atlantic salmon (42). First, we verified that, similar to salmonids, the ILT in adult zebrafish contains a similar network of reticulated epithelial cells (**Figure 7A**). Then, examination of this lymphoid structure at the base of filaments following a cytokeratin labeling revealed a striking network of reticulated epithelial cells (**Figure 7B**), indicating that this is a new structured gill lymphoid tissue. Based on its localization along each side of the gill arches, we named it the Amphibranchial Lymphoid Tissue (ALT). Of note, the ALT sometimes reached the efferent aspect of the filament at the ventral end of the gill arch (**Figure S2C**). The localizations of the ILT and ALT within the gill arch are schematized **Figure 8**.

Lymphoid tissues require access to nutrients, oxygen, and ways to facilitate immune cell trafficking to maintain their functions. Thus, we looked for the presence of endothelial structures close to the ALT. Cryosections through non-dissected gills from fli:GFP adult zebrafish (n = 3), whose endothelial cells are green fluorescent, revealed that the ALT is in contact with the first basal lamellae of filaments (**Figures S4A, B** cyan arrows). In addition, we also observed the presence of endothelial cells in the connective tissue close to the ALTs (**Figure S4B** cyan arrowheads). Wholemount acquisitions of dissected gill arches revealed that these endothelial vessels sometimes sprouted from the edge of the most basal lamellae and, at times, formed anastomoses (cyan star) connecting with the efferent artery of the next filament (**Figure S4C** green arrowheads). Further work will be required to determine if these endothelial vessels are blood vessels, lymphatic vessels, or the hybrid vascular/lymphatic vessels such as those described by Burne in anglerfish and cod (71, 72). Such hybrid vessels, called “fine vessels” by Burne, display a morphology similar to arteries but have not been reported in zebrafish to date.

### The ILT and ALT Share Features of Secondary Lymphoid Organs and Are Not Additional Thymi

In our effort to further characterize the zf-GIALT, we investigated putative role of the zebrafish ILT and ALT as T/NK cell enriched areas. In a recent investigation, Koppang et al. found evidence that the salmonid ILT is not an additional thymus (73) but most likely secondary lymphoid in nature (74). To facilitate the induction of immune responses, secondary lymphoid organs also contain many myeloid cells, in particular professional antigen-presenting cells. Therefore, we looked for the immune cell types that in addition to T/NK cells, compose the ILT and ALT.

**Figure 9:**
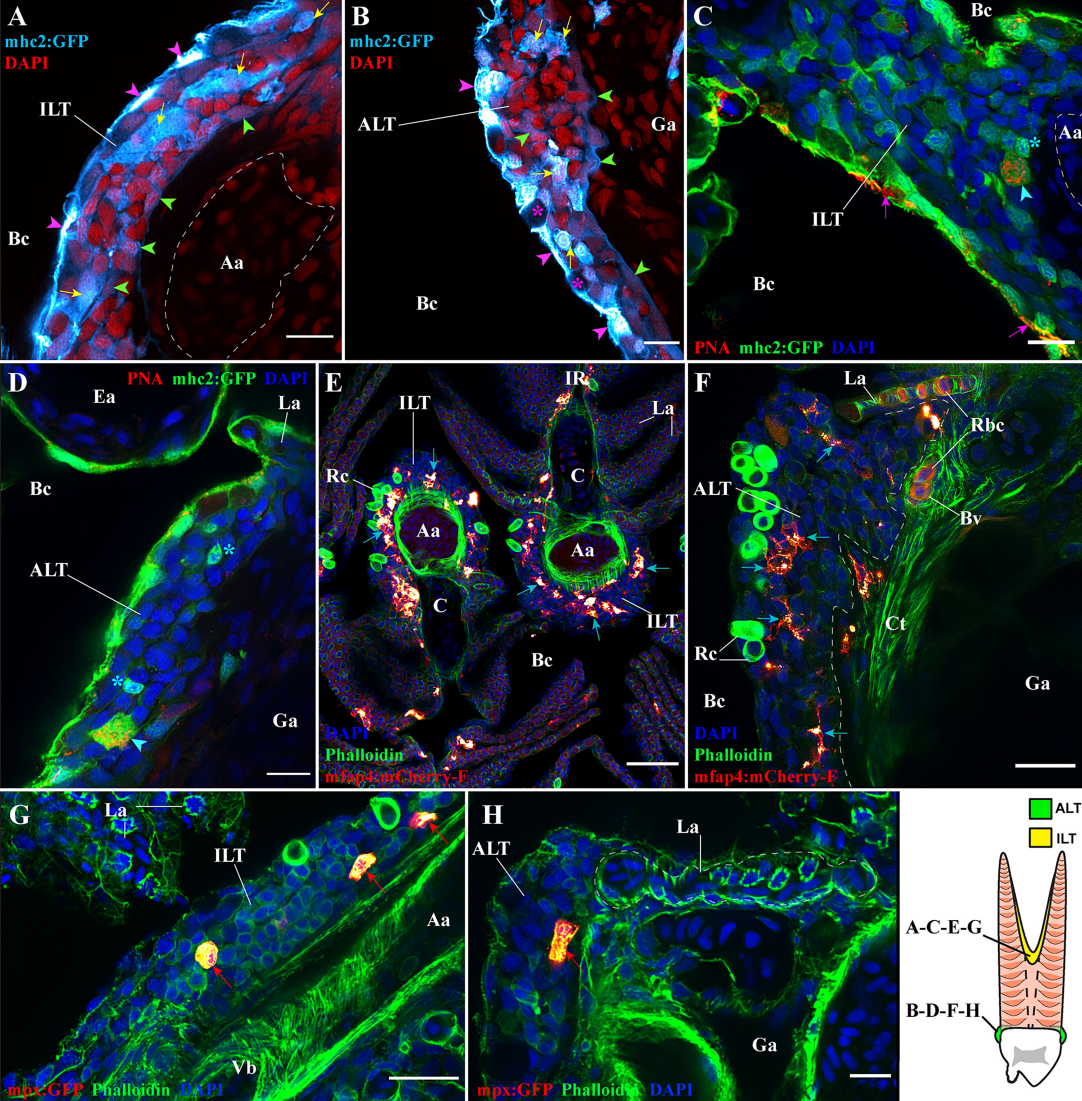
Antigen-presenting cells and granulocytes are also present within the ILT and ALT. Representative deconvolved confocal images of adult zebrafish gills displaying the ILT **(A, C, E, G)** and the ALT **(B, D, F, H)** at high-magnification, as illustrated on panel **(I)**. Images were acquired from 30 μm whole-body cryosections. Cryosection showing the ILT **(A)** and ALT **(B)** of mhc2:GFP (cyan hot) zebrafish stained with DAPI (red) reveal numerous *mhc2* expressing cells. Purple arrowheads point to GFP positives pavement cells, green arrowheads highlight GFP low reticulated epithelial cells and yellow arrows indicate the presence of large GFP positive cells. **(C, D)** Cryosections from mhc2:GFP (green) zebrafish gills, stained with fluorescent peanut agglutinin lectins (red) and DAPI (blue), show the presence of dendritic cells among the ILT and ALT (cyan arrowheads), and the presence of small GFP^+^ cells displaying a lymphocyte-like morphology (cyan stars). To note, the peanut agglutinin lectin also binds to the surface of some pavement cells (magenta arrows). **(E, F)** Cryosections from mfap4:mCherry-F zebrafish, whose macrophages are fluorescent, stained with phalloidin (green) and DAPI (blue), identify the presence of many macrophages (red hot) within the ILT and ALT (cyan arrows). **(G, H)** Cryosections from mpx:GFP zebrafish, whose neutrophils are fluorescent, stained with phalloidin (green) and DAPI (blue), identify the presence of neutrophils (red hot) within the ILT and ALT (red arrows). Images are maximum intensity projections: 2 µm **(A–D, G, H)**, 4 µm **(F)** and 9 µm **(E)**. Annotations: Aa, Afferent artery; ALT, Amphibranchial Lymphoid Tissue; Bc, Branchial cavity; C, Cartilage; Ea, Efferent artery; Ga, Gill arch; ILT, Interbranchial Lymphoid Tissue; La, Lamella; Rbc, Red blood cells; Rc, Rodlet cells and Vb, Vascular bleb. Scale bars: 10 µm **(A–D, H)**, 20 μm **(F, G)** and 40 µm **(E)**.

We first investigated the presence of antigen-presenting cells in ILT and ALT of adult zebrafish using the mhc2:GFP transgenic line where mhc2^+^ cells are green fluorescent (n =3). We observed a strong expression of GFP by pavement cells (keratinocytes) (magenta arrowheads) and by mucous cells (magenta stars) of the gill epithelium (**Figures 9A, B**). GFP expression by keratinocytes (pavement cells) in fish older than 45 days has been previously reported by the creators of this reporter line (15) and is consistent with the expression of *mhc2* by gill epithelial cells as previously observed in Atlantic salmon (75). In the deeper layers of the epithelium, we noticed the presence of weakly labeled GFP cells (green arrowheads) that form a meshwork and compose the basal layer of the ILT (**Figure 9A**) and ALT (**Figure 9B**). Co-labeling with anti-cytokeratin identified these cells as reticulated epithelial cells (not shown). This is consistent with the suggested expression of *mhc2* by thymic reticulated epithelial cells (15). Importantly, we also found many large cells (yellow arrows), as well as smaller ones displaying a lymphocyte-like morphology (cyan stars), among the GFP high cells from both ILT and ALT, suggesting professional antigen-presenting cells may also be abundant in these lymphoid tissues (**Figures 9A–D**).

In support, we stained sections from mhc2:GFP zebrafish with a red fluorescent peanut agglutinin as described in (16), confirming the likely presence of dendritic cells (**Figures 9C, D** cyan arrowheads) (n =3). Using the mfap4:mCherry-F zebrafish line that labels macrophages (60), we found the presence of many macrophages in both the ILT and ALT (**Figures 9E, F** cyan arrows) (n = 3). Finally, granulocytes such as neutrophils, were also present in both lymphoid tissue (**Figures 9G, H** red arrows) (n = 3). Interestingly, mhc2:GFP positive cells and ZAP70 positive cells were frequently observed adhering to the luminal side of the vascular bleb endothelium beneath the ILT (**Figure S5** Cyan arrowheads and Yellow arrowheads).

**Figure 10:**
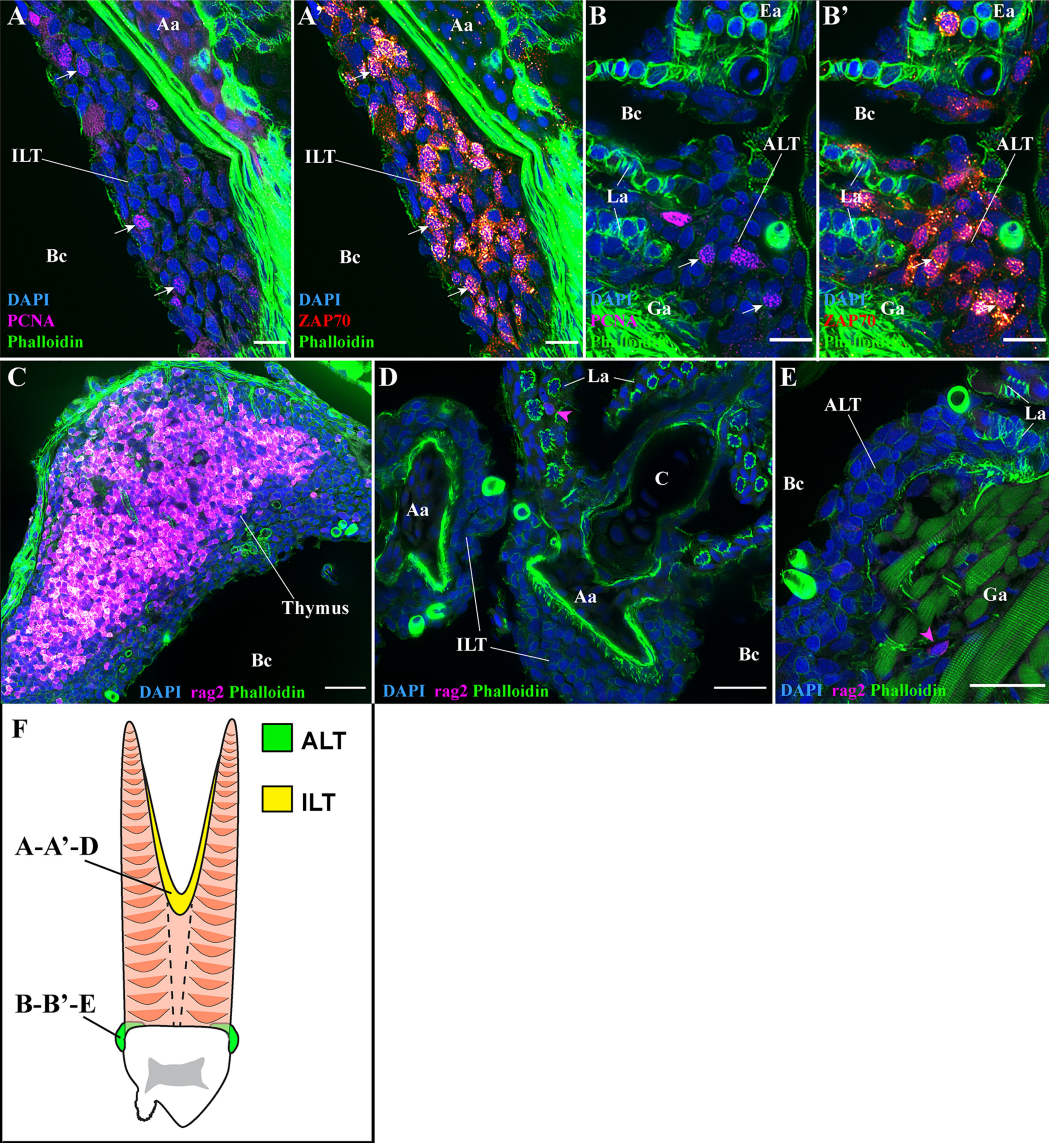
T/NK cell proliferation and RAG2 expression. Representative deconvolved confocal images of adult zebrafish gills displaying the ILT **(A, A’, D)**, the ALT **(B, B’, E)**, as illustrated on panel **(F)**, and the thymus **(C),** at high-magnification. Images were acquired from 30 μm whole-body cryosections stained with phalloidin (green) and DAPI (blue). **(A, B’)** Co-labeling of T/NK cells with an anti-ZAP70 antibody (red hot), and of proliferating cells with an anti-PCNA antibody (magenta). Proliferating T/NK cells in the ILT and ALT are highlighted by white arrows in **(A, A’, B, B’)**. **(C–E)** Cryosections from rag2:DsRED zebrafish where cells going through V(D)J recombination are fluorescent (magenta hot). Expression of rag2 is very high within the thymus but only a few cells with low signal are present around the ILT and ALT (magenta arrowheads in **(D, E)**. Images are maximum intensity projections: 2 µm **(A–E)**. Annotations: Aa, Afferent artery; ALT, Amphibranchial Lymphoid Tissue; Bc, Branchial cavity; C, Cartilage; Ea, Efferent artery; Ga, Gill arch; ILT, Interbranchial Lymphoid Tissue and La, Lamella. Scale bars: 10 µm **(A, B’)**, 20 μm **(D, E)** and 30 µm **(C)**.

To further explore if the ALT and ILT displayed features of secondary lymphoid organs, we combined staining with anti-ZAP70 with an anti-PCNA labeling, a marker of proliferative cells. The results showed that T/NK cells proliferate in both the ILT and the ALT of healthy adult zebrafish (**Figures 10A, B’** white arrows) (n = 3). Since T cell proliferation also occurs in the thymus, we next looked for the expression of the protein RAG2, a marker of antigen recombination highly expressed by thymocytes. Imaging of rag2:DsRED zebrafish cryosections revealed that in sharp contrast to the high number of RAG2-positive cells in the thymus (**Figure 10C**), RAG2-positive cells were rare in the ILT and ALT, indicating that these lymphoid structures are not additional thymi (**Figures 10D, E** magenta arrowheads) (n = 3). Collectively, our zebrafish data are in agreement with previous studies that showed the presence of T cell proliferation and the absence of T cell antigen recombination in the Atlantic salmon ILT (42, 73), highlighting a certain degree of conservation between those two distant fish species.

Altogether, these data indicate that the zebrafish ILT and ALT, the two structured sub-regions of the zf-GIALT, do not represent additional thymi but share several features of secondary lymphoid organ.

### The zf-GIALT Is Subjected to Rapid Structural Modifications During Infection With the Spring Viraemia of Carp Virus

We next investigated if the zf-GIALT is affected by a severe systemic viral infection. For this, we used the Spring Viraemia of Carp Virus (SVCV) rhabdoviral infection model of the adult zebrafish. Identified in 1971 (65), SVCV is a negative-sense single-stranded RNA virus that is responsible for highly lethal infections in cyprinids (76), including zebrafish (30, 77). The gills are believed to be a possible portal of entry for SVCV, before its dissemination to internal organs such as the head-kidney, liver, spleen and brain (78, 79). Upon natural or bath infections, SVCV induces severe pathological changes including gills paleness, inflammation, necrosis, hyperplasia, and hemorrhages (30, 76, 80). Adults zebrafish were infected by immersion in a suspension of SVCV (10^4^ pfu/ml) for 48 hours, sampled at 3 days and 10 days post-infection (dpi), and processed for high-resolution imaging. Immunofluorescence with anti-SVCV-N antibody confirmed that the fishes were infected (**Figure S6**). All specimens from the infected group displayed important amounts of SVCV-N throughout the organism. The virus was found in the blood, the meninges, brain tissues, the cerebrospinal fluid, the liver, the head-kidney and in connective tissues.

Gills are another important site of SVCV infection. Here, numerous cells with SVCV-N signal were found within and around the afferent arch artery (**Figure S6H** white and yellow arrowheads) and within the septum (magenta arrowheads). Other major features of gill infection were the presence of numerous highly infected cells, with fragmented nuclei, contained within the lamellae blood circulation (**Figures S6H, I** red arrowheads and **Supplementary Video S9**), and a high SVCV-N signal by the filament cartilage and interlamellar regions (**Figure S6H–J** cyan arrowheads). In individuals collected 10 dpi, damage to the gills was more pronounced, with the presence of occasional necrosis and hyperplasia (**Figure S6K** yellow arrowheads). Interestingly, besides the intense infection status of the gills, we only detected little to moderate SVCV-N signal in the ALT and ILT (**Figures S6L, M** cyan arrowheads), that could be attributed to infection or antigen internalization. This striking disparity in SVCV-N distribution among the gills and these structured lymphoid sub-regions of the zf-GIALT, is of particular interest in regard to their possible involvement in the defense of gills against pathogens. This finding is coherent with a previous study (81) that observed important replications of infectious salmon anaemia virus (ISAV) in gills of bath-infected Atlantic salmons but detected no viral replication within the ILT.

**Figure 11:**
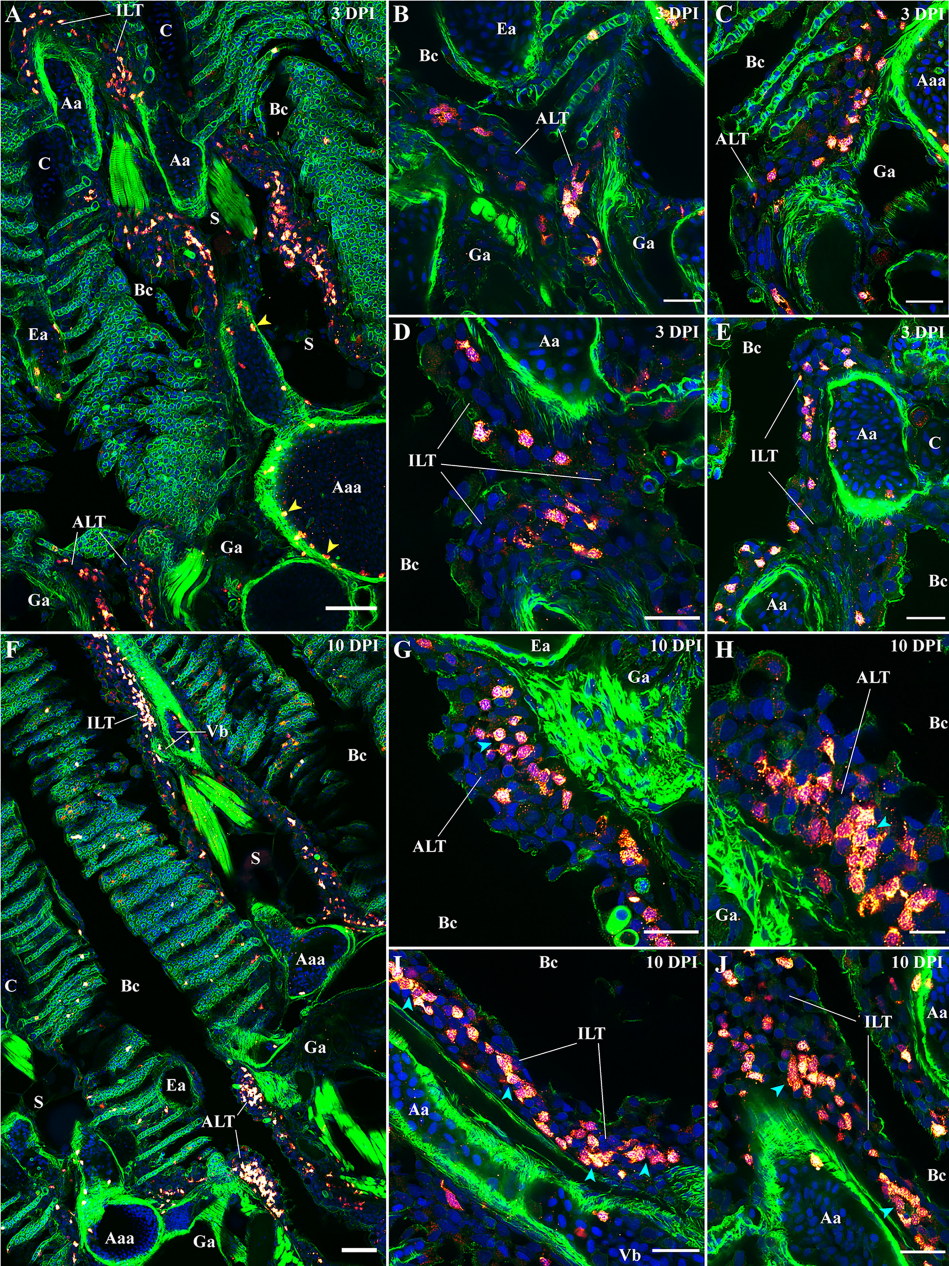
Modifications of the zf-GIALT structure upon SVCV infection. Representative deconvolved confocal images of adult zebrafish gills 3 days **(A–E)** and 10 days **(F–J)** post-infection with the Spring Viraemia of Carp Virus (SVCV). Images were acquired from 30 μm whole-body cryosections stained with phalloidin (green) and DAPI (blue) and where T/NK cells were labeled with anti-ZAP70 antibody (red hot). **(A)** Low magnification image of a gill arch illustrating the overall reduced number of T/NK cells in the gills 3 days after infection. This depletion of T/NK cells is particularly striking within the ALT **(B, C)** and the ILT **(D, E)**. Yellow arrowheads in **(A)** point to T/NK cells adhering to the endothelium of blood vessels. **(F)** Low magnification image of two gill arches illustrating the replenishment of the zf-GIALT 10 days after infection. The number of T/NK cells within the ALT **(G, H)** and the ILT **(I, J)** is a lot higher; and T/NK cells form small clusters (cyan arrowheads in **H**, **I** and **J**). Images are maximum intensity projections: 2 µm **(B–E, G–J)** and 5 µm **(A, F)**. Annotations: Aa, Afferent artery; Aaa, Afferent arch artery; ALT, Amphibranchial Lymphoid Tissue; Bc, Branchial cavity; C, Cartilage; Ea, Efferent artery; Ga, Gill arch; ILT, Interbranchial Lymphoid Tissue; La, Lamella; S, Septum and Vb, Vascular bleb. Scale bars: 10 µm **(H)**, 20 μm **(B–E, G, I, J)** and 50 µm **(A, F)**.

For the next step, we used the anti-ZAP70 antibody to investigate the zf-GIALT organization at 3 and 10 dpi **(** **Figures 11F–J**). On 3 dpi with SVCV (**Figures 11A–E**), we observed a strong reduction of the number of T/NK cells from all 5 sub-regions of the zf-GIALT compared to uninfected fish (**Figures 2** **–** **6**). Both the ALT and ILT shared an even more striking reduction in T/NK cells (**Figures 11B–E**). These structured lymphoid tissues were reduced in size and T/NK cells were no longer the dominant cell type. Furthermore, the clustering of the ZAP70+ cells was also lost. These observations are consistent with a previous study showing shrinkage of the Atlantic salmon ILT upon infection with the virus VHSV (73).

This significant decrease in T/NK cells populating the zf-GIALT raises several questions: Are these cells disappearing because of infection-related cell deathor do they migrate elsewhere in the body? T/NK cells have not been described as targets for SVCV and we did not observe infection of ZAP70+ cells by the virus. Besides, the decrease of T/NK cells in ILT and ALT coincided with the appearance of high numbers of ZAP70+ cells within blood vessels throughout the organism, often seen in contact with the endothelium (**Figure 11A** yellow arrowheads). Further investigation will be required to determine if some of these circulating T/NK cells emigrated from the zf-GIALT. A week later, at 10 dpi, many more T/NK cells were again present within the zf-GIALT (**Figure 11F**). In addition to an increase of ZAP70+ cells being associated with lamellae, both the ALT and the ILT appeared larger and displayed numerous T/NK cells again (**Figures 11G–J**). This replenishment of the ALT and ILT coincided with new clustering of T/NK cells (Cyan arrowheads) surrounded by remaining ZAP70- cells. Collectively, our data show that the zf-GIALT undergoes complex structural changes upon SVCV infection, starting with a marked reduction in T/NK cells in both the ILT and ALT after 3 dpi, followed by a rapid and striking replenishment of the ILT and ALT with clustering of T/NK cells at 10 dpi. These dynamic T/NK cell reactions of the ILT and the ALT are compatible with their possible involvement in immune responses, further strengthening the hypothesis that they represent secondary lymphoid structures. However, many crucial questions remain open: Are the micro-clusters of T/NK cells corresponding to antigen-induced clonal expansion? Are they the formation of T cell zones? Are the ZAP70- cells belonging to B-cell lineages?

Overall, our study provides novel evidences supporting the presence of secondary lymphoid structures in zebrafish gills with at least two sub-regions, the ILT and the ALT. However, a definitive identification would require detailed investigations of the lymphocytes phenotypes, their activation markers and their clonal expansion in response to antigens.

### The ALT Is a Preserved Lymphoid Tissue Across Distant Teleost Families

**Figure 12:**
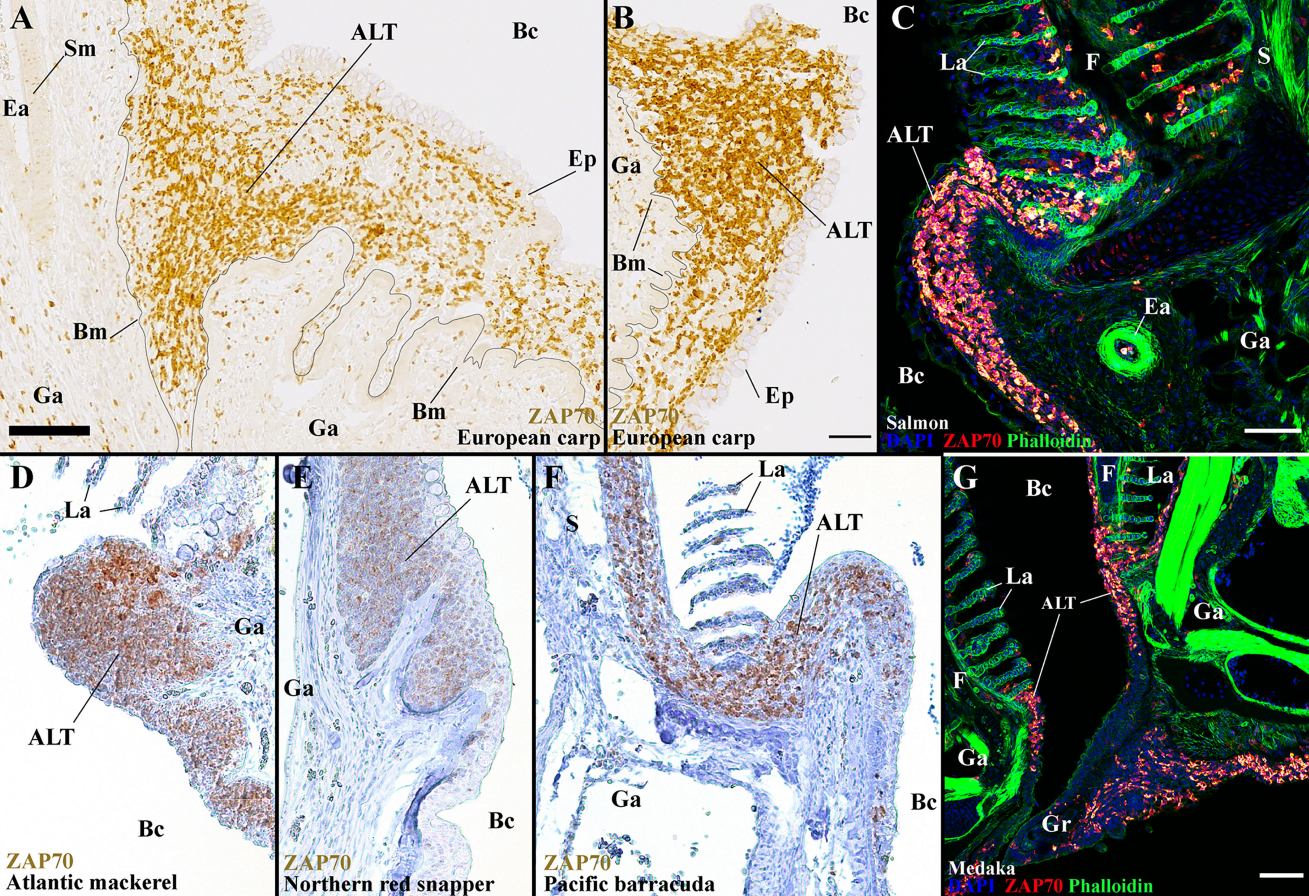
The ALT is a preserved structure between distant teleost species. Representative acquisitions of anti-ZAP70 (brown/red hot) labeled gills section, from different fish species, displaying the side of a gill arch, at the base of filaments. **(A, B)** ALT of the European carp (*Cyprinus carpio*); Cyprinid – Paraffin section. **(C)** ALT of the Atlantic salmon (*Salmo salar L.*); Salmonid – cryosections stained with phalloidin (green) and DAPI (blue). **(D)** ALT of the Atlantic mackerel (*Scomber scombrus*); Scombridae, Percomorph – Paraffin section. **(E)** ALT of the Northern red snapper (*Lutjanus campechanus*); Lutjanidae, Percomorph – Paraffin section. **(F)** ALT of the Pacific barracuda (*Sphyraena argentea*); Sphyraenidae, Percomorph – Paraffin section. **(G)** ALT of the Medaka (*Oryzias latipes*); Adrianichthyidae, Percomorph - cryosections stained with phalloidin (green) and DAPI (blue). These images are maximum intensity projections: 2 µm **(G)** and 5 µm **(C)**. Annotations: ALT, Amphibranchial Lymphoid Tissue; Bc, Branchial cavity; Bm, Basement membrane; Ea, Efferent artery; Ep, Epithelial cells; F, Filament; Ga, Gill arch; Gr, Gill raker; La, Lamella; S, Septum and Sm, Smooth muscles. Scale bars: 50 µm **(B, G)** and 70 μm **(A, C)**.

In this study, we have characterized the zf-GIALT in detail and discovered a new structured lymphoid tissue at the base of the filaments, the ALT. Our results suggest that both the ILT and ALT participate in the gill defenses against pathogens. In a recent study (44), we found that the ILT was present among “basal” teleosts, despite great size variations between species, but appeared absent in “modern” teleost species (*i.e.*, Percomorphs). We now wondered if the ALT would share a similar evolutionary pattern of presence or absence since the anti-ZAP70 antibody targets a highly conserved epitope among teleost species (44), allowing us to perform a comparative research. Our first step was to study the presence of ALT in another member of the cyprinid family, the European common carp (*Cyprinus carpio*). Similar to zebrafish, carp gills displayed prominent ALTs at the base of their filaments (**Figures 12A, B**). Since adult carps are around a thousand times larger than a zebrafish, the presence of an ALT apparently is independent of size variations between fish species. Then, we studied the gills of Atlantic salmon (*Salmo salar L.*), a representative of the salmonids (another group of “basal” teleosts). Salmon gills arches also display a prominent ALT (**Figure 12C**). Of particular interest, while we could not detect a clear ILT among percomorphs in our previous study, we found a clear ALT in several species from this large taxonomic group including Atlantic mackerel (*Scomber scombrus*), Northern red snapper (*Lutjanus campechanus*), Pacific barracuda (*Sphyraena argentea*), and Medaka (*Oryzias latipes*) (**Figures 12D–G**). In species such as European perch (*Perca fluviatilis*) and Witch flounder (*Glyptocephalus cynoglossus*), the T/NK cell aggregates at the basis of the gill filaments in (44) actually correspond to ALT. The presence of ALT across all examined teleosts, but the apparent loss of ILT in “modern” fish, constitutes an interesting enigma.

## Discussion

The organization of the mucosal immune system in mammals is thought to support efficient antigen trapping and rapid activation of the adaptive immune response (82). Organized-MALT (O-MALT) in mammals include Peyer’s patches in the gut and tonsils in the naso-pharyngeal cavity. These O-MALT structures constitute the inductive sites, where the adaptive immune response is initiated. Cells from these inductive sites migrate to the effector sites, for instance, the lamina propria of the gut. The distinction between inductive and effector mucosal sites is still blurry (83) in teleosts due to the fact that the teleost mucosal immune system has always been thought of being diffuse (d-MALT). However, lack of description of O-MALT structures in teleosts may be due to limited reagents used to label immune cells as well as to sub-optimal imaging approaches that miss the 3-D complexity of mucosal tissues such as the gills.

Our study exemplifies how 3-D high-resolution imaging can help uncover the organization of the mucosal immune system of teleosts. We adopted an approach combining immunology, histology and advanced imaging to dissect the structural organization of the gill-associated lymphoid tissue (zf-GIALT). Our study unveiled a complex compartmentalization into five sub-regions that are associated with different morphological territories within the gills and show variable abundance and organization of T/NK cells. Our high-resolution image descriptions confirmed the presence of three sub-regions where zf-GIALT is diffuse (d-GIALT), while the remaining two regions constitute structured lymphoid aggregates (evoking O-GIALT). Our most significant finding is the discovery of a structured lymphoid tissue along the sides of the gill arches, a sub-region that we called the Amphibranchial Lymphoid Tissue (ALT), which constitutes one of the two O-GIALT sub-regions. Like the ILT, despite the presence of high numbers of T/NK cells, the ALT is not an additional thymus, since RAG2 expression level is very low, but it shares several features associated with secondary lymphoid structures. Importantly, upon infection of adult zebrafish with the rhabdovirus SVCV, zf-GIALT underwent significant structural changes, suggesting the involvement of both, ILT and ALT, in immune responses. Finally, where the ILT was absent from some (neo)teleost species, the ALT region appears to have adapted to various fish families and lifestyles.

The present study illustrates the power of advanced imaging to link the localization of molecular immune markers to its cell spatial context. The combination of the optical advantages of the zebrafish model with a modern spinning-disk confocal microscope made possible the investigation of large biological volumes at high-resolution. In the absence of dissection, we achieved an optimal preservation of the biological context (e.g. connective tissues, vascular system, boundaries between organs, etc), which allowed us to explore all four pairs of gill arches simultaneously. Analysis at the whole organism level was also important in our characterization of the impact of SVCV infection. Along this line, the optical investigation of the immune system at the tissue and organism level at high-resolution is currently gaining importance (84, 85).

The gill is a vital organ that is highly exposed to foreign elements and pathogens. The demonstration of an acquired and pathogen-specific immunity associated to gills was first described by Reuling a hundred years ago (53). Reuling studied the reaction of the fish to the parasitic larval stage of mussels that attaches to gills, the Glochidia. He showed that successive gill infections of large-mouth black bass (*Micropterus salmoides*) with Glochidia of the mussel *Lampsilis luteola* induced a specific immunity that was not related to mechanical factors, such as mucus clearance, but to a humoral blood factor.

To date, our understanding of gill immunology remains largely incomplete and the mechanisms of local acquired immunity are not well understood. So far, most studies investigating the teleost gill immune system have addressed the gill organ as a whole, as if it had a homogeneous structure. This is particularly true regarding the zebrafish model in which gill populations of Th2-like T cells (86), foxp3a^+^ regulatory T cells (87), and gill-associated metaphocytes (57) have been identified though not specifically localized in gills sub-regions. Further, candidate immune-related gene expression at the level of whole (dissected) gills (56, 86, 88) has made significant progress but lacks spatial transcriptional resolution. Overall, the lack of consideration for the complex architecture of the gills is problematic as the organization of the zebrafish gill immune system is largely heterogeneous. Indeed, our study shows that T/NK cell distribution within each compartment is variable. Within the interlamellar region, the efferent aspect of filaments, as well as within the septum and the gill arch, T/NK cells are scattered. In contrast, both the ILT and ALT correspond to structured lymphoid tissues where numerous T/NK cells are embedded in a complex scaffold of reticulated epithelial cells. It therefore appears highly relevant to link gill immune functions and markers, to the different regions of the gills, especially true for the ILT and ALT. The expression of certain immune-related genes may be much more pronounced in the ILT or ALT, where immune cells are dominant, than in the other regions of the gills. Such new analyses would lead to a more dynamic and spatial understanding of immune-related gene expression and local immune responses. This is highly relevant for immune responses to water-borne infections, and for the distribution of certain cell-types in the gills.

Our discovery of a hitherto non-described lymphoid tissue underscores the fact that the fish immune system is structurally sophisticated and may encourage for a new assessment of the commonly used terminology for mucosal immune tissues in fish. Previous studies have confirmed the participation of the salmon ILT during the course of an immune response to viral infection (73). Here, infection of adult zebrafish with the rhabdovirus SVCV revealed that the zf-GIALT undergoes significant structural changes. Our time course experiment critically revealed dynamic changes in the T/NK cell populations of the zf-GIALT and indicate the involvement of both the ALT and ILT in this infection model. Future studies may identify whether T/NK cells are actually proliferating and migrating out of the ILT and ALT early during infection. Further, the fate of the T/NK cells that exit the ILT and ALT in response to infection requires careful future examination. While our data suggest the ALT is likely involved in immune responses, it is possible that they play a role in interactions between immune cells and the local microbiota. Following infection with SVCV, we noticed a striking increase of circulating T/NK cells in the blood while the ALT showed a dramatically reduced amount of T/NK cells. Investigations of cell trafficking between the structured lymphoid tissues of the zf-GIALT and the rest of the organism, including other MALT, will likely provide new insights regarding immune cell circulation and inductive and effector sites in teleosts. Interestingly, a thickening and lck^+^ T cell invasion of the distal ILT (dILT) and filaments of gills in adult zebrafish where IL-10 (56) and foxp3a (21) are knocked out, strongly suggests that tight regulation is required *in situ*, possibly to avoid an overstimulation of the GIALT.

The ALT and ILT share many similarities, but their phylogenetic distribution differs. The percomorph species we analyzed had typical ALT but no ILT, while the other teleosts had both ALT and ILT. These observations confirm our previous report (44) in which we did not find ILT in several percomorphs including the European perch and the Witch flounder, while these species displayed lymphoid aggregates at the base of filaments corresponding to ALT. In percomorph, gills do not have interbranchial septa and the filaments are separated by a wider angle than in “basal” teleost branches. This highlights the need to address how morphological adaptations constrain the evolution of immune tissues. We note that one percomorph, the southern bluefin tuna (*Thunnus Maccoyii*) has been reported in a Ph.D. thesis to possess both an interbranchial septum and an ILT (89), calling for more comparative analyses to understand the evolution of gill immune tissues in this very large group of teleosts. On a wider perspective, regarding the evolution of mucosal immunity, one may ask whether the ILT and/or the ALT represent a (functional) counterpart of the Waldeyer’s ring that protects the nasopharyngeal cavity of mammals (90, 91).

From a more applied perspective, the zebrafish model is widely used by multiple scientific communities to study diseases affecting humans, and also farmed fish (6–8, 92). Our data provide an opportunity to analyze a number of zebrafish disease models from a different point of view. For example, Feng et al. studied the role of bcl-2 in the dissemination of T-Lymphoblastic Lymphoma Cells in adult zebrafish (93), and observed large aggregates of cancer cells within the gills, which were particularly prominent in the ILT and at the gill arch. In this context, understanding cell trafficking and functions of ALT and ILT immune cells would lead to new questions regarding interactions between tumor cells and their environment, especially the mechanisms of invasion of lymphoid tissues.

Finally, ILT and ALT biology is clearly important to better understand a number of diseases of farmed (and wild) fish. Gill diseases represent an increasingly important burden, but the immune mechanisms they trigger remain poorly known. Our findings could lead to new advances in host-pathogen interactions, such as the ones involving sea-lice, a devastating parasite for the salmon industry that infect the gills and skin (94), and they may help understanding “Complex gill diseases”, an emerging syndrome caused by various gill pathogens (95). Considering the potential role of gills as a portal of entry for pathogens, and toxic compounds, it is therefore important to improve our knowledge of the gill lymphoid tissues, and how they are affected by mechanical treatments such as thermal delousing.

## Conclusion

Altogether, our study enabled a better understanding of the gill immune system that emphasizes its structural heterogeneity. A significant finding for fish immunology is the discovery of a new structured lymphoid tissue in the gills of adult zebrafish and other teleost species, which we have named the Amphibranchial Lymphoid Tissue (ALT). We show that ALT and ILT are not additional thymi, but rather share important features of secondary lymphoid organs, and likely play an important role in acquired immunity. These findings are important for understanding gill health and mucosal immunity, and are therefore relevant both for aquaculture and for developing fish models of infections and pathologies.

## Data Availability Statement

The original contributions presented in the study are included in the article/**Supplementary Material**. Further inquiries can be directed to the corresponding author.

## Ethics Statement

The animal study was reviewed and approved by COMETHEA (INRAE Research Center) and the French Ministry of Agriculture (authorization number APAFIS# 22150-2019062811528052).

## Author Contributions

Conceptualization: GW, EK, IS, PB, and JR. Investigation: AD, AK, SK, ED, DR, AL-P, and JR. Methodology: AD, AK, HB, ED, and JR. Validation: JR. Writing - Original draft: GG, PB, and JR. Writing - Review & Editing: AD, AK, ED, DR, HB, GG, GW, EK, IS, PB, and JR. Visualization: AD, AK, AL-P, and JR. Supervision: GG, GW, EK, IS, PB, and JR. Project administration: JR. All authors contributed to the article and approved the submitted version.

## Funding

We thank the Norwegian Research Council for funding (FRIMEDBIO-No 144642). In addition, PB was supported by the VetBioNet project (EU Grant Agreement INFRA-2016-1 731014) and by institutional grants from INRAE.

## Conflict of Interest

The authors declare that the research was conducted in the absence of any commercial or financial relationships that could be construed as a potential conflict of interest.

The reviewer FT declared a past co-authorship with one of the author IS to the handling editor at the time of review.

## Publisher’s Note

All claims expressed in this article are solely those of the authors and do not necessarily represent those of their affiliated organizations, or those of the publisher, the editors and the reviewers. Any product that may be evaluated in this article, or claim that may be made by its manufacturer, is not guaranteed or endorsed by the publisher.

## Abbreviations

Aa, Afferent artery; Aaa, Afferent arch artery; Af, Afferent aspect of filaments; ALT, Amphibranchial Lymphoid Tissue; Am, Adductor muscles; Bc, Branchial cavity; C, Cartilage; dILT, distal Interbranchial Lymphoid Tissue; Ea, Efferent artery; Eaa, Efferent arch artery; Ef, Efferent aspect of filaments; F, Filament; Ga, Gill arch; GALT, Gut-Associated Lymphoid Tissue; GIALT, Gill-Associated Lymphoid Tissue; d-GIALT, (Diffuse) Gill-Associated Lymphoid Tissue; O-GIALT, (Organized) Gill-Associated Lymphoid Tissue; ILT, Interbranchial Lymphoid Tissue; IR, Interlamellar Region; ISAV, Infectious Salmon Anaemia Virus; La, Lamellae; M, Muscles; MALT, Mucosa-Associated Lymphoid Tissue; d-MALT, (Diffuse) Mucosa-Associated Lymphoid Tissue; O-MALT, (Organized) Mucosa-Associated Lymphoid Tissue; Mc, Mucous cell; NALT, Nasal-Associated Lymphoid Tissue; pILT, proximal Interbranchial Lymphoid Tissue; Pvc, Pavement cell; Rbc, Red blood cells; Rc, Rodlet cell; S, Septum; SALT, Skin-Associated Lymphoid Tissue; Sm, Smooth muscles; SVCV, Spring Viraemia of Carp Virus; Sw, Septum wall; Tb, Taste bud; Vb, Vascular bleb (ampulla); VHSV, Viral Hemorrhagic Septicemia Virus; Vs, Venous sinus; Zf-GIALT, Zebrafish Gill-Associated Lymphoid Tissue.
