# Supplementary material for: High-Resolution, 3D Imaging of the Zebrafish Gill-Associated Lymphoid Tissue (GIALT) Reveals a Novel Lymphoid Structure, the Amphibranchial Lymphoid Tissue
Source: Front Immunol. 2021 Nov 16;12:769901. doi: 10.3389/fimmu.2021.769901 (PMC8647647; doi:10.3389/fimmu.2021.769901)
Supplement: Supplementary file 1 [file DataSheet_1.pdf]

Supplementary Material

Supplementary Figures

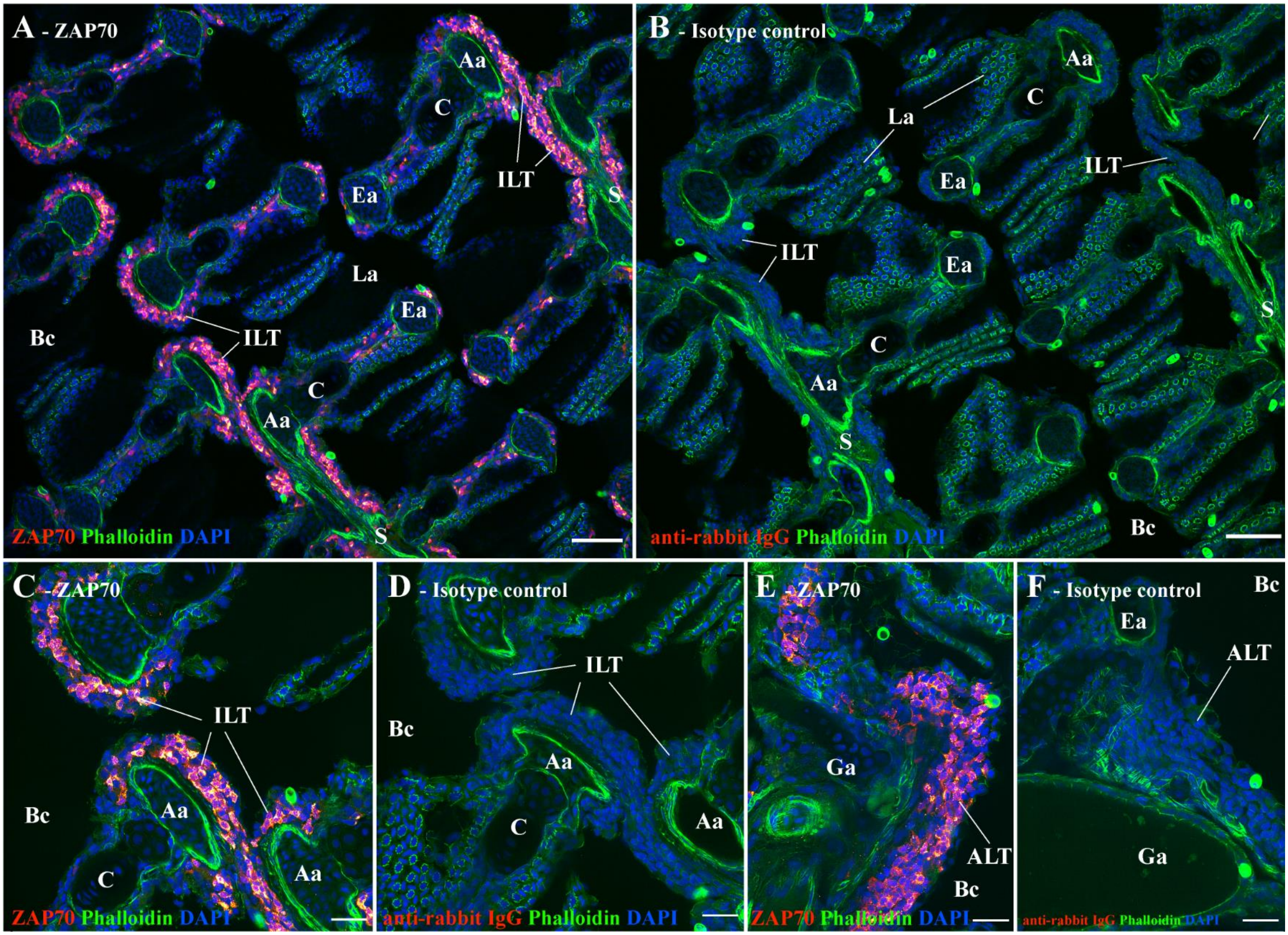

Figure S1. Anti-rabbit IgG isotype control.

Representative deconvolved confocal images from 30 µm cryosections of adult zebrafish branchial cavity stained with phalloidin (green), DAPI (blue), and either rabbit anti-ZAP70 **(A, C, E)** or rabbit IgG isotype control **(B, D, F)** (red hot) at 0,5 µg/mL**.** The images display the ILT at low **(A-B)** and high **(C-D)** magnifications, and the ALT at high magnifications **(E-F)**. The images are maximum intensity projections (< 2 µm). Annotations: Aa, Afferent artery; ALT, Amphibranchial Lymphoid Tissue; Bc, Branchial cavity; C, Cartilage; Ea, Efferent artery; Ga, Gill arch; ILT, Interbranchial Lymphoid Tissue; La, Lamellae and S, Septum. Scale bar: 20 µm **(C-F)** and 50 µm **(A-B)**.

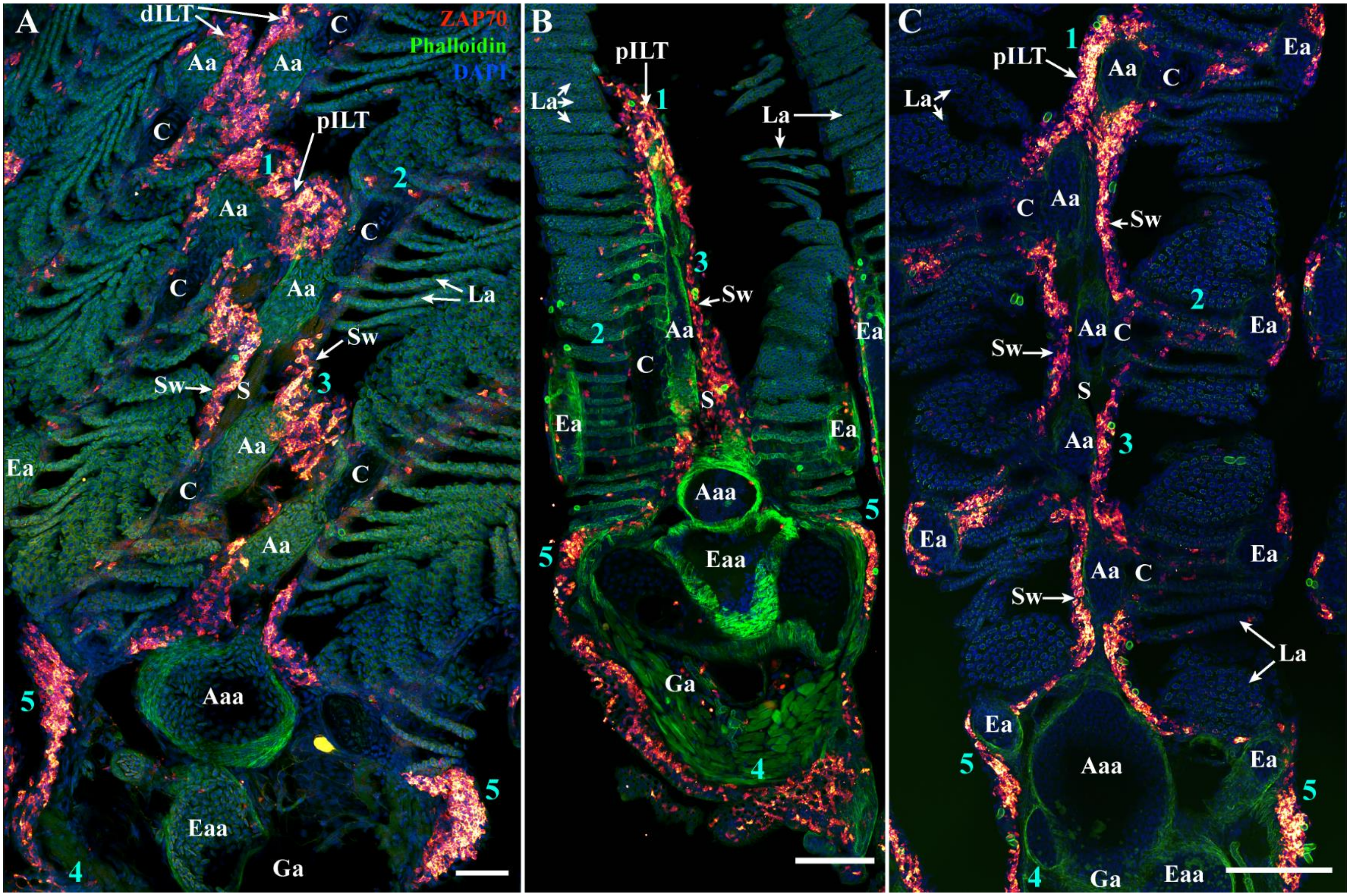

Figure S2. General organization of the zebrafish GIALT, other specimens.

Representative deconvolved confocal images of gills from 3 adult zebrafish acquired from an oblique longitudinal **(A,C)** and transversal orientations **(B)**. Images were acquired from 30 µm whole-body cryosections stained with phalloidin (green) and DAPI (blue) and where T / NK cells are labeled with anti-ZAP70 antibody (red hot). The gills from the 3 different specimens displayed a distribution of ZAP70 positive cells similar to the **Figure 1** with a segmentation into five sub-regions (1-5) (ILT, interlamellar region-lamellae-efferent aspect of filaments, interbranchial septum, gill arch, T cell clusters at the base of filaments on each side of the gill arch). **(A-C)** Images are maximum intensity projections (MIP). Annotations: Aa, Afferent artery; Aaa, Afferent arch artery; Bc, Branchial cavity; C, Cartilage; dILT, distal Interbranchial Lymphoid Tissue; Eaa, Efferent arch artery; Ea, Efferent artery; F, Filament; Ga, Gill arch; Gr, Gill raker; La, Lamellae; pILT, proximal Interbranchial Lymphoid Tissue; S, Septum; Sw, Septum wall. Scale bars: 50 µm **(A)** and 100 µm **(B,C)**.

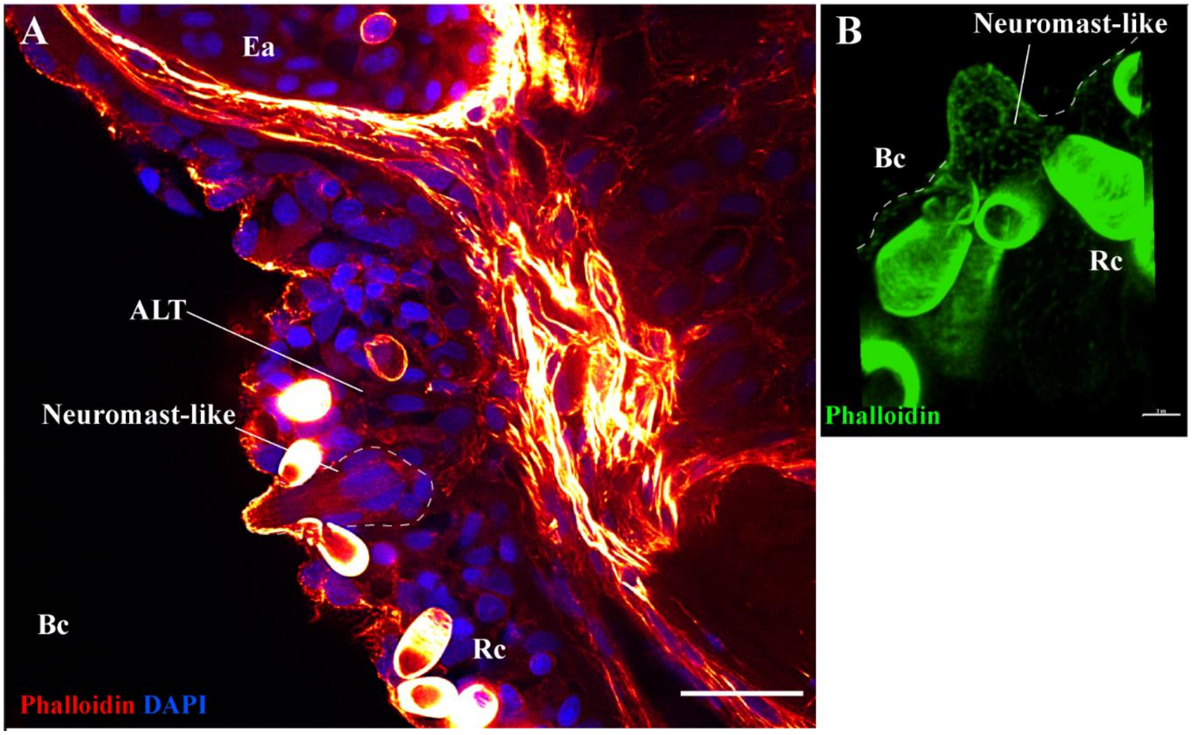

Figure S3. Neuromast-like structures in the ALT.

(A) Representative deconvolved confocal image of an adult zebrafish ALT with transversal orientation The image was acquired from a 30 µm whole-body cryosections stained with phalloidin (red hot/green) and DAPI (blue). Highlighted by the dotted line is the neuromast/taste-bud like structure found in the ALT. **(B)** Image from a 3D reconstruction of the ALT surface showing the protrusion formed by the neuromast/taste-bud like structure. **(A)** The image is a maximum intensity projections (2 µm). Annotations: ALT, Amphibranchial Lymphoid Tissue; Bc, Branchial cavity; Ea, Efferent artery and Rc, Rodlet cells. Scale bar: 3 µm **(B)** and 20 µm **(A)**.

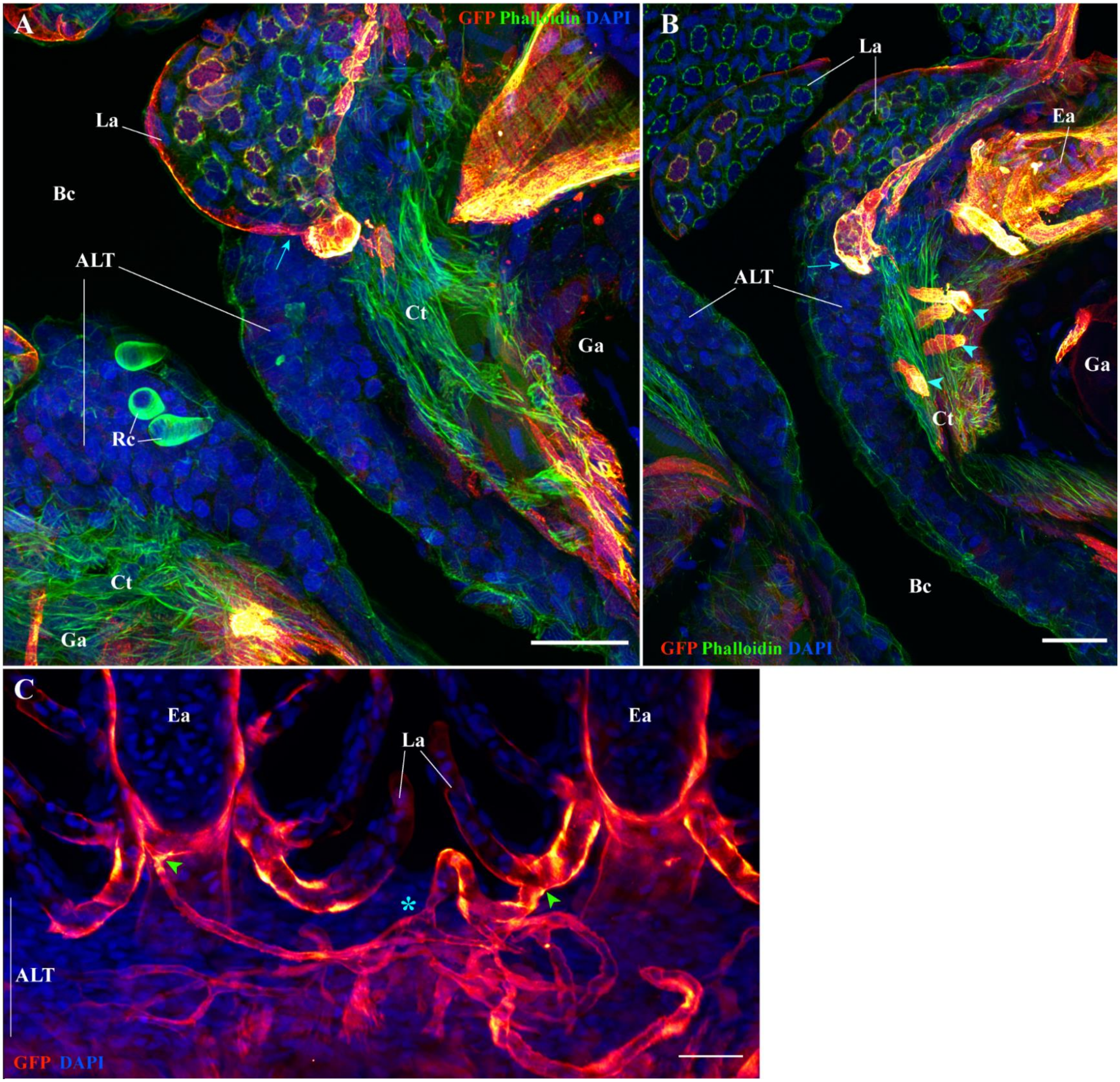

Figure S4. Close proximity of the ALT with vascular compartments.

Representative deconvolved confocal high-resolution images of fli:GFP adult zebrafish ALT acquired with a transversal **(A,B)** and sagittal orientations **(C)**. Images were acquired from 30 µm whole-body cryosections stained with phalloidin (green) and DAPI (blue). Endothelial cells from blood and lymphatic vessels express GFP (Red hot). **(A,B)** The ALT is in direct contact with the vascular compartment of the most basal lamellae of each filaments (cyan arrows). In addition, the ALT is close to many vessels located on the nearby connective tissue (cyan arrowheads). **(C)** Occasionally, the ALT lie on anastomotic vessels (cyan star) that connect two successive efferent arteries (green arrowheads). Images are maximum intensity projections. Annotations: ALT, Amphibranchial Lymphoid Tissue; Bc, Branchial cavity; Ct, Connective tissue; Ea, Efferent artery; Ga, Gill arch; La, Lamellae and Rc, Rodlet cells. Scale bars: 20 µm **(A-C)**.

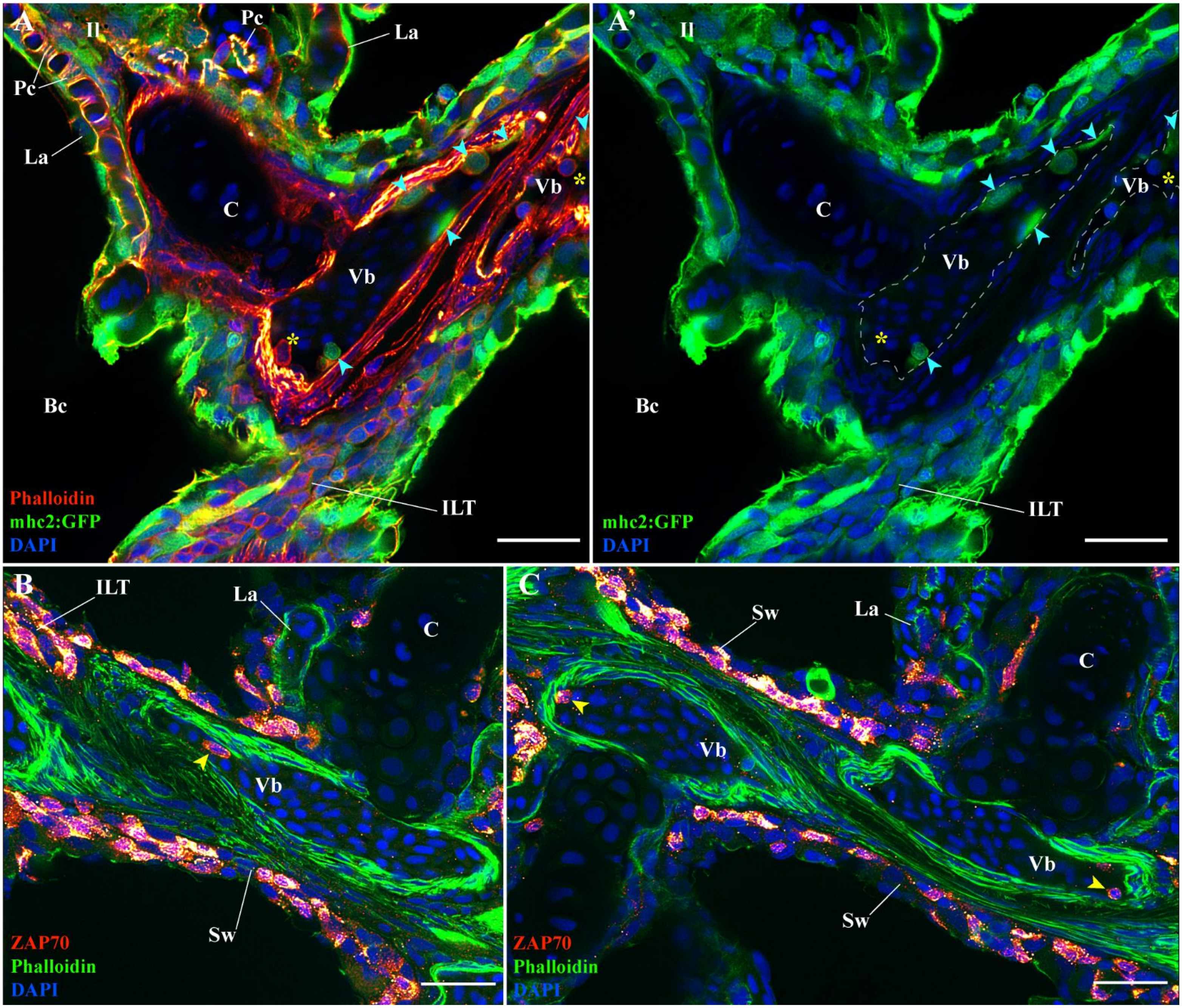

Figure S5. Adhesion of immune cells to the endothelium of vascular blebs.

Representative deconvolved confocal high-resolution images of adult zebrafish gills acquired with a coronal orientation. **(A-A')** 30 µm whole-body cryosection from mhc2:GFP (green) zebrafish stained with phalloidin (Red hot) and DAPI (blue). Many mhc2 expressing cells bind to the endothelium of the vascular bleb (cyan arrowheads), right beneath the ILT. Some actin-rich GFP negative circulating cells also adhere to the endothelium of vascular blebs (yellow stars). **(B-C)** 30 µm whole-body cryosections from wild-type zebrafish stained with phalloidin (green) and DAPI (blue), and where T / NK cells are labeled with anti-ZAP70 antibody (red hot). ZAP70 positive cells adhering to the endothelium of the vascular blebs are frequent observed (yellow arrowheads). Images are maximum intensity projections: 2 µm **(A-C)**. Annotations: Bc, Branchial cavity; C, Cartilage; Eaa, Efferent arch artery; IL, Interlamellar region; ILT, Interbranchial Lymphoid Tissue; La, Lamellae; Pc, Pillar cells; Sw, Septum wall and Vb, Vascular bleb. Scale bars: 20 µm **(A-C)**.

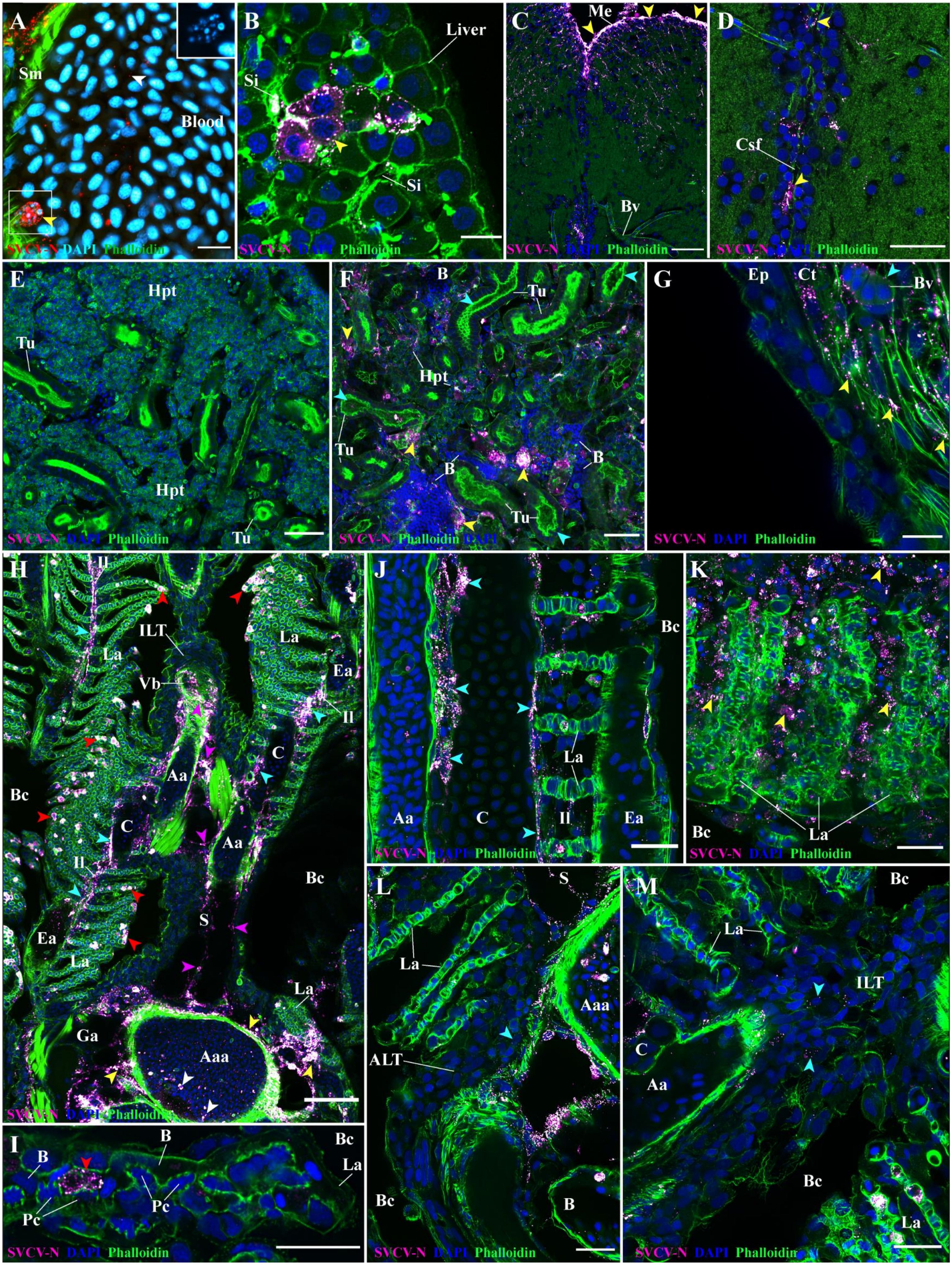
A
Sm
Blood
SVCV-N DAPI Phalloidin
B
Liver
Si
Si
SVCV-N DAPI Phalloidin
C
Me
Bv
SVCV-N DAPI Phalloidin
D
Csf
SVCV-N DAPI Phalloidin
E
Hpt
Tu
Hpt
Tu
SVCV-N DAPI Phalloidin
F
B
Tu
Hpt
Tu
B
B
Tu
SVCV-N Phalloidin DAPI
G
Ep
Ct
Bv
SVCV-N DAPI Phalloidin
H
Il
ILT
La
Vb
La
Ea
Il
C
Aa
Bc
C
Aa
Il
Bc
S
Ea
La
La
Ga
Aaa
SVCV-N DAPI Phalloidin
I
B
B
Bc
La
Pc
Pc
SVCV-N DAPI Phalloidin
J
Bc
La
Aa
C
Il
Ea
SVCV-N DAPI Phalloidin
K
Bc
La
SVCV-N DAPI Phalloidin
L
S
La
La
Aaa
ALT
Bc
B
SVCV-N DAPI Phalloidin
M
Bc
La
ILT
C
Aa
Bc
La
SVCV-N DAPI Phalloidin

Figure S6. Distribution of SVCV infection sites.

Representative deconvolved confocal images of an adult zebrafish infected with SVCV. The images were acquired from 30 µm whole-body cryosections. Unless specified, the sections were stained with phalloidin (green) and DAPI (blue), and SVCV particles were labeled with anti-SVCV-N antibody (magenta hot). **(A)** Afferent arch artery of an infected fish showing circulating infected cells (red) with fragmented nuclei (cyan hot) (yellow arrowhead) and circulating viral particles (white arrowhead). Although only a few endothelial cells bear an anti-SVCV-N signal, a high SVCV-N signal was found in the connective tissue beyond the smooth muscle layers of major blood vessels. **(B)** SVCV infection of hepatocytes (yellow arrowhead). **(C)** SVCV infection of the brain and meninges (yellow arrowheads) **(D)** SVCV infection of the cerebrospinal fluid (yellow arrowheads). **(E)** Head-kidney of a healthy adult zebrafish. **(F)** In infected fish, the head-kidney was heavily infected (yellow arrowheads)and suffered severe damages such as reduced hematopoietic tissues and altered tubules (cyan arrowheads). Some of the highly infected cells expressed the macrophage marker MPEG1 (not shown). **(G)** Illustration of the strong infection of connective tissues throughout the organism (yellow arrowheads). **(H-M)** Distribution of SVCV particles in the gills. SVCV particles are abundant in the connective tissue of the gills arch (**H** - yellow arrowheads), within the numerous infected cells contained within the blood flow of the lamellae (**H,I** – red arrowheads), in the connective tissue surrounding the cartilage of filament (**H,J** - cyan arrowheads), within the interbranchial septum (**H** – magenta arrowheads) and in hyperplasia/necrotic regions of the lamellae (**K** – yellow arrowheads). In contrast, few SVCV particles are found in the ALT (**L** – cyan arrowhead) and ILT (**M** – cyan arrowheads). Images are maximum intensity projections: 0,5 µm **(I)**, 2 µm **(A-G,J-M)** and 5 µm **(H)**. Annotations: Aa, Afferent artery; Aaa, Afferent arch artery; ALT, Amphibranchial Lymphoid Tissue; B, Blood; Bc, Branchial cavity; Bv, Blood vessel; C, Cartilage; Csf, Cerebrospinal fluid; Ct, Connective tissue; Ea, Efferent artery; Ep, Epithelium; Ga, Gill arch; Hpt, Hematopoietic tissue; Il, Interlamellar region; ILT, Interbranchial Lymphoid Tissue; La, Lamellae; Me, Meninges; Pc, Pillar cells; S, Septum; Si, Sinusoid; Sm, Smooth muscles; Tu, Tubules and Vb, Vascular bleb. Scale bar: 10 µm **(B)**, 20 µm **(A,G,I-M)**, 30 µm **(D)**, 50 µm **(E,F)** and 70 µm **(C)**.

Supplementary videos:

Supplementary videos are available in Figshare. DOI: 10.6084/m9.figshare.16558497

For an optimal quality, we recommend to download the files to watch them.

Video S1. Organization of the zf-GIALT from a single branchial cavity.

Video of a representative 3D deconvolved confocal image of an adult zebrafish branchial cavity displaying the four gill arches with a transversal orientation. The video was acquired from a 30 µm whole-body cryosections stained with phalloidin (red) and DAPI (blue), and where T / NK cells are labeled with anti-ZAP70 antibody (white). At the beginning of the video, the pharynx is at the bottom, the skin at the top, teeth to the left and the mouth is toward to right.

Video S2. Interbranchial lymphoid tissue

Video of a representative 3D deconvolved confocal image of an adult zebrafish proximal interbranchial lymphoid tissue seen with a coronal orientation. The video was acquired from a 30 µm whole-body cryosections stained with phalloidin (red) and DAPI (blue), and where T / NK cells are labeled with anti-ZAP70 antibody (white). Note the contrast of the ZAP70 labeling between the interbranchial lymphoid tissue in the middle and the rest of the filaments.

Video S3. Organization of the zf-GIALT at the gill arch

Video of a representative 3D deconvolved confocal image of an adult zebrafish gill arch seen with a transversal orientation. The video was acquired from a 30 µm whole-body cryosections stained with phalloidin (red) and DAPI (blue), and where T / NK cells are labeled with anti-ZAP70 antibody (white). Note the presence of the two T cell cluster on the sides of the gill arch, at the base of filaments.

Video S4. Organization of the zf-GIALT at the pharyngeal side of the gill arch.

Video of a representative 3D deconvolved confocal image of an adult zebrafish pharyngeal side of a gill arch seen with a transversal orientation. The video was acquired from a 30 µm whole-body cryosections stained with phalloidin (red) and DAPI (blue), and where T / NK cells are labeled with anti-ZAP70 antibody (white). Note the presence of the two T cell clusters on the sides of the gill arch, at the base of filaments.

Video S5. Vascular bleb

Video of a representative 3D deconvolved confocal image of a fli:GFP adult zebrafish vascular blebs seen with a transversal orientation. The video was acquired from a 30 µm whole-body cryosections stained with phalloidin (green) and DAPI (blue), and where endothelial cells are fluorescent (red hot). The video goes through the optical sections of the 3D acquisition. The vascular bleb are at the bottom right and are located at the uppermost region of the septum. The vascular blebs represent radial enlargement of the afferent artery of each filament. Note that the vascular bleb from two successive filaments does not directly connect with each other's. Lamellae are seen at the bottom right of the video, only the cells on the edge of the vascular compartment express GFP.

Video S6. Vascular bleb does not connect with each other's but forms radial pits.

Video of a representative 3D deconvolved confocal image of a fli:GFP adult zebrafish vascular blebs seen with a transversal orientation. The video was acquired from a 30 µm whole-body cryosections stained with phalloidin (green) and DAPI (blue), and where endothelial cells are fluorescent (red hot). The video goes through the optical sections of the 3D acquisition. The vascular bleb is at the middle left of the video, to the right is an afferent artery. Through the optical sections, the vascular bleb is getting closed, indicating it is forming a pit and not a tube directly interconnecting with the vascular blebs of other filaments.

Video S7. 3D architecture of the amphibranchial lymphoid tissue.

Video of a representative 3D deconvolved confocal image of an adult zebrafish gill arch seen with a sagittal orientation. The video was acquired from a wholemount gill arch stained with phalloidin (red) and DAPI (blue), and where T / NK cells are labeled with anti-ZAP70 antibody (white). The ALT is a continuous lymphoid structure along the sides of gill arches. The ALT is at its thinnest at the level of efferent arteries and at its thickest when in-between filaments. At the start of the video, the gill rakers are at the bottom and the filaments are at the top. To note an interesting morphological peculiarity, a filament to the right is segmented in two.

Video S8. Neuromast/taste-bud like structure of the ALT.

Video of a representative 3D deconvolved confocal image of an adult zebrafish ALT displaying a neuromast/taste-bud like structure. The video was acquired from a 30 µm whole-body cryosections stained with phalloidin (green) and DAPI (blue). These structures are protruding from the ALT in a similar fashion as taste-buds and neuromasts.

Video S9. Highly SVCV infected cell contained in the lamellae blood compartment.

Video of a representative 3D deconvolved confocal image of an SVCV infected adult zebrafish lamellae displaying an infected cells contained in the blood of a lamellae. The video was acquired from a 30 µm whole-body cryosections stained with phalloidin (green) and DAPI (blue), and where SVCV particles are labeled with an anti-SVCV-N antibody (red hot). These highly infected cells have a fragmented nuclei and appears stuck between pillar cells, which are the cells displaying a ring like phalloidin pattern.
